# A Fuzzy Logic-Based Directional Charging Scheme for Wireless Rechargeable Sensor Networks

**DOI:** 10.3390/s24155070

**Published:** 2024-08-05

**Authors:** Yuhan Ma, Chao Sha, Yue Wang, Jingwen Wang, Ruchuan Wang

**Affiliations:** School of Computer Science, Software and Cyberspace Security, Nanjing University of Posts and Telecommunications, Nanjing 210003, China; mayuhan1219@163.com (Y.M.); b21111203@njupt.edu.cn (Y.W.); wjw1072045262@163.com (J.W.); wangrc@njupt.edu.cn (R.W.)

**Keywords:** wireless rechargeable sensor networks, directional charging, fuzzy logic system, charging efficiency, staying point optimization

## Abstract

Wireless Power Transfer (WPT) has become a key technology to extend network lifetime in Wireless Rechargeable Sensor Networks (WRSNs). The traditional omnidirectional recharging method has a wider range of energy radiation, but it inevitably results in more energy waste. By contrast, the directional recharging mode enables most of the energy to be focused in a predetermined direction that achieves higher recharging efficiency. However, the MC (Mobile Charger) in this mode can only supply energy to a few nodes in each direction. Thus, how to set the location of staying points of the MC, its service sequence and its charging orientation are all important issues related to the benefit of energy replenishment. To address these problems, we propose a Fuzzy Logic-based Directional Charging (FLDC) scheme for Wireless Rechargeable Sensor Networks. Firstly, the network is divided into adjacent regular hexagonal grids which are exactly the charging regions for the MC. Then, with the help of a double-layer fuzzy logic system, a priority of nodes and grids is obtained that dynamically determines the trajectory of the MC during each round of service, i.e., the charging sequence. Next, the location of the MC’s staying points is optimized to minimize the sum of charging distances between MC and nodes in the same grid. Finally, the discretized charging directions of the MC at each staying point are adjusted to further improve the charging efficiency. Simulation results show that FLDC performs well in both the charging benefit of nodes and the energy efficiency of the MC.

## 1. Introduction

In Wireless Sensor Networks (WSNs), a large number of cheap microsensor nodes are deployed in a specific area to form a multi-hop and self-organized network for monitoring [1]. However, due to the limited battery capacity of nodes, the network is always energy-constrained. That is to say, when some nodes die, an energy hole will inevitably appear which has a significant impact on the functionality of the network [2].

Fortunately, Kurs et al. [3] demonstrated that energy can be transferred between magnetically resonant coils in a strongly coupled regime, and a number of Wireless Power Transfer (WPT) techniques have been developed, such as magnetic resonant coupling, inductive coupling, and electromagnetic radiation [4]. By utilizing the WPT technology to provide wireless, reliable, and continuous energy supply for sensors, it promotes the development of Wireless Rechargeable Sensor Networks (WRSNs) which has a wide range of research prospects for wearable devices [5], access control authentication [6] and Industrial Internet of Things [7,8].

In WRSNs, one or more Mobile Chargers (MCs) are responsible for supplying energy to nodes periodically or on demand [9]. Obviously, nodes which will run out of energy should be replenished in time. Thus, it is particularly important for the MC to set an appropriate order to serve nodes, which is regarded as the “charging scheduling problem” [10].

There are mainly two types of charging scheduling schemes in WRSNs, i.e., periodical charging [11] and on-demand charging [12,13]. In the former mode, the MC periodically replenishes energy to nodes, and the duration for a round of charging can be either fixed or adjustable. During each round, the MCV sets off from the Base Station (BS) and traverses some nodes in a predefined order to recharge them. Finally, it goes back to the BS for a quick battery replacement and prepares for the next round of recharging services. Obviously, this charging method is simple and easy to implement, but it cannot adapt to changes in energy demand of nodes. In on-demand charging, the charging request is sent out when a node’s energy falls below a certain threshold. This request is placed into the service queue of the MC according to a predetermined priority [14]. When receiving the recharging requests from nodes, the MCV departs from the BS as soon as possible and starts its energy replenishment service. Then, it recharges these nodes according to the calculated order and returns to the BS when its residual energy is insufficient. Obviously, this mode is more suitable for WRSNs, but it is always a key issue to set the priority of nodes being replenished.

In addition, according to different mechanisms of energy radiation, the replenishment mode for the MC can also be categorized into omnidirectional charging and directional charging. An MC with omnidirectional WPT broadcasts electromagnetic waves equally in all directions. Since the radiated energy fades rapidly over distance, chargers might require excessively high transmission power to supply enough energy to nodes. By contrast, in directional charging mode, energy can be concentrated in a narrow beam for transmission, which greatly improves the charging efficiency in WRSNs [15]. However, in most of the existing works, charging orientations of the MC are simply determined by the geometric location of each node as well as the energy radiation region of the MC. It does not consider dynamic information such as the residual energy or the charging demand of nodes, which is precisely one of the problems that we need to solve in this paper.

To address the issues raised above, we propose a Fuzzy Logic-based Directional Charging (FLDC) scheme. As we all know, fuzzy logic systems are flexible, user friendly, easy to understand, and an important tool for decision-making, so they can be very helpful for dynamic charging scheduling planning [16,17]. The main contributions of this paper are summarized as follows.

- Considering the fact that the MC often charges directionally in each discrete orientation at each staying point, the network in FLDC is divided into a number of virtual grids. Then, to ensure that nodes can be fairly recharged, we propose a method for setting the priorities of each grid based on a two-layer fuzzy logic system. Thus, a reasonable order for the MC to traverse each staying point is obtained.
- In addition, the locations of the staying points are further optimized based on the gradient descent method. This shortens the average distance between the nodes to be recharged and the MC, thus effectively reducing the energy loss during charging.
- Based on the priority of nodes to be recharged, each discrete charging direction of the MC as well as the rotational order of its charge orientations at each staying point are sequentially calculated. Furthermore, to balance the energy receiving efficiency of each node within the same energy radiation range, the charging direction of the MC is fine-tuned, thereby effectively shortening the charging duration.

The rest of this paper is organized as follows. In Section 2, we show the current research status at home and abroad in this field and some typical algorithms for directional charging scheduling in WRSNs. The network structure is introduced in Section 3, and related parameter settings as well as the problem description are discussed in Section 4. In Section 5, we describe the fuzzy logic-based directional charging scheme in detail. Simulation results are shown in Section 6, and the conclusions of this method are summarized in Section 7.

## 2. Related Works

In this section, we review the related works according to the two categorization methods mentioned above.

### 2.1. Periodic Charging and On-Demand Charging

In periodic charging, the MC usually replenishes energy to nodes following a fixed trajectory round by round. Obviously, in a scenario where the energy consumption rate of nodes is constant, this model has some advantages. That is, once the path for the MC to charge nodes is established, it does not need to be generated again. Xie et al. [18] considered a scenario where mobile chargers periodically visit all nodes in a sensor network and charge them to achieve sustainable network operation. They proposed a pre-optimized TSP-based charging protocol to achieve this goal. However, this may lead to unnecessary visits to nodes with sufficient energy. This not only increases the movement distance of the MC, but also may prolong the waiting time for nodes that urgently need to be recharged. To address this issue, Hu et al. [19] proposed an efficient slot-based periodic charging time scheduling algorithm. They adopted a fine-grained node classification scheme to prevent unnecessary visits to energy-sufficient nodes. Moreover, a balanced charging task assignment scheme was also used in this method to avoid charging starvation.

To avoid node deaths, many periodic charging methods assume that the MC has sufficient or even an infinite amount of energy, but this is not realistic. For this reason, Huong et al. [20] proposed a periodic charging scheme for WRSNs with the goal of minimizing the number of dead nodes, considering the limited battery capacity of the MC. In addition, they presented a two-phase algorithm to obtain an optimal charging path for the MC, as well as the charging duration for each node. In one of our previous works [21], a periodic and distributed energy supplementation method was proposed with the help of a ring-based cost-balanced data uploading strategy. It performs well at enhancing the proportion of live nodes as well as the wireless recharging efficiency. Moreover, Lyu et al. [22] divided the network into multiple cells, and the MC periodically traverses each cell with the objective of maximizing the amount of data collected as well as its charging efficiency.

Relatively speaking, the on-demand charging mode is more adaptable to large-scale networks. Quan et al. [23] introduced an on-demand charging scheme based on Q-learning, which maximizes the survival rate of nodes while ensuring network connectivity as well as sensing coverage. Locations of all the critical nodes are calculated, and they are then recharged in a way that improves the performance of the whole network. Similarly, in [24], locations of all nodes that can be served in time are first calculated. Next, the most appropriate charging duration for various staying points can be obtained. This ensures that the next node being recharged by the MC is always the optimal target. In addition, Lin et al. [25] proposed a temporal-spatial real-time charging scheduling algorithm (TSCA) for the on-demand charging mode to prolong network lifetime by maximizing recharging efficiency. TSCA achieved feasible solutions by using node insertion and deletion algorithms based on their locations relative to the MC and the subsequent node.

Recent research increasingly focuses, in on-demand charging mode, on dividing the network into several areas to allow multiple MCs to supply energy to nodes in a distributed manner, thereby improving energy efficiency. Lin et al. [26] proposed a dual warning threshold with dual preemption (DWDP) charging scheme, which uses dual warning thresholds to adjust the charging priority of different sensors when the residual energy level of a sensor node is lower than a certain threshold. Then, DWDP is extended to the scenario of multiple mobile chargers, and a cooperative charging method CCDWDP is proposed. Tomar et al. [27] thought that most of the on-demand charging schemes leave out the contemplation of multiple network attributes while making scheduling decisions and even overlook the issue of ill-timed charging response to nodes with uneven energy consumption rates. Thus, they proposed a fuzzy logic-based charging scheduling method that assigns the MCs to *k* different regions. Furthermore, the dynamic recharging threshold is individually set for each node. However, collaboration among multiple MCs is not considered in this method. Yang et al. [28] proposed a multi-type charging scheduling strategy based on the difference of area requirement. The network is divided into inner and outer regions. Nodes in the inner region form a flat topology, and a periodic and one-to-one charging mode is adopted by a mobile charger to maintain a high survival rate of nodes. In contrast, nodes located in the outer region form several clusters, and multiple UAVs supply energy to these nodes in a one-to-many manner to achieve higher energy efficiency.

### 2.2. Omnidirectional Charging and Directional Charging

Nowadays, the omnidirectional charging mode is widely adopted in scenarios with dense node deployment. Ma et al. [29] proposed a solution to the problem of maximizing charging utility when multiple nodes are charging simultaneously. The main constraint is the battery capacity of the mobile charger. They also studied the problem of minimizing the length of an MC’s movement path under the assumption that all nodes which require charging can be recharged and the mobile charger has sufficient energy to do so. To address the problem of slow wireless charging speed affecting network operational efficiency, Xu et al. [30] proposed a many-to-many energy replenishment strategy based on the omnidirectional charging mode. Experimental results show that this method effectively shortens the waiting duration for nodes to be recharged. Dande et al. [31] proposed an efficient energy recharge scheduling for MCs, aiming to maximize the spatial and temporal surveillance qualities (STSQs) of the given network. Firstly, the network is partitioned to distribute the MC’s charging load evenly. Then, the sensors adjust the sensing rate frequency to manage their energy. Finally, cooperation between the neighboring MCs is proposed to reduce the waiting time for recharging requested sensors. However, they did not provide further explanation on how to balance the workload of multiple MCs.

Compared to omnidirectional charging, the energy radiation model of directional charging is more realistic. Thus, more and more researchers have studied the charging scheduling problem based on the directional charging mode. Wang et al. [32] proposed a radio-frequency-based WPT in which a base station with multiple directional antennas is responsible for charging nodes. However, in this model, the position of the base station is fixed, so it cannot be applied to large-scale networks. In [33], Lee et al. proposed an energy-efficient directional charging (EEADC) algorithm that considers the density of sensor nodes to adaptively choose one-to-one or one-to-many charging modes. With the help of directional antennas, the optimal charging orientation for an MC can be calculated out in EEADC, thereby minimizing its energy loss during the charging process. Gao et al. [34] pointed out that the existing research neglects cooperative energy re-distribution (ERD) among nodes for energy charging efficiency. Therefore, they focused on the scenario of charging a WRSN using a directional mobile charger and addressed the underlying ERD-assisted directional charging schedule problem. Moreover, Wu et al. [35] studied cooperative scheduling for directional charging with spatial occupation. They formulated a cooperative charging scheduling model with spatial occupation problem of mobile rechargeable sensor devices for optimizing the total cost of charging the whole system.

Unlike the above studies that deployed only a single type of MC, the deployment of a mixture of directional and omnidirectional mobile chargers is considered by Lin et al. [36]. They proposed a method that can find the minimum number of chargers needed to cover all the sensor nodes. Moreover, the energy radiation power of the charger is also taken into consideration when determining the traversal position of the MC. Wang et al. [37] proposed an Average Energy Charging (AEC) algorithm. Firstly, directed coverage subsets of the network with large increases in charging utility are selected. Then, locations of staying points as well as the movement path of the MC can be obtained with the help of these subsets. Next, the charging duration during a round of service is evenly distributed to each coverage subset to balance the charging utility of them. In addition, they further proposed a Maximum Utility Charging (MUC) algorithm to improve the performance of AEC by assigning an appropriate charging duration to each subset based on the energy replenishment requirement of nodes located in this region.

The related work presented above is summarized in Table 1.

In this paper, we chose AEC and MUC as the comparison methods for the following reasons.

- Both AEC and MUC adopt a periodic charging scheduling method, which is consistent with the service mode of the MC proposed in this paper;
- Both AEC and MUC address issues such as determining the charging orientation of the MC, forming several coverage subsets to enable the MC to perform one-to-many charging in order, path planning, etc., which are also the focus of this paper.

## 3. Network Model

Assuming that *N* nodes (each of them is denoted as *S_i_* (*i*∈[1, *N*])) are randomly deployed in a rectangular network with length *L* and width *W*. Each of the nodes is equipped with a magnetically coupled coil that can be wirelessly recharged, and all of them have full battery capacity, i.e., *E_s_*, at the beginning of the network running time. The base station is located at any fixed position within the network, and it is responsible for the following tasks.

- Collecting and processing all the sensing data.
- Scheduling and controlling the energy replenishment behavior of each MC.
- Replacing the battery for MCs with insufficient residual energy.

**Figure 1:**
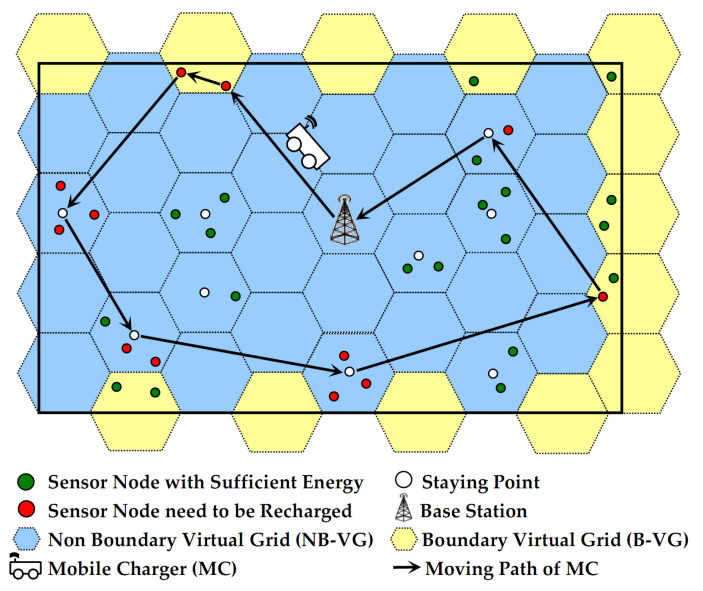
Network architecture.

The network model is shown in Figure 1.

**Figure 2:**
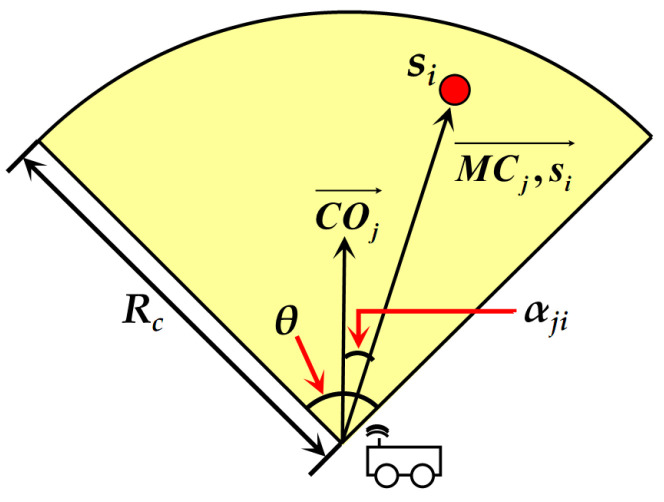
Energy radiation range of the MC.

There are *N_MC_* MCs in the network (the calculation process of *N_MC_* is described in Section 4.1), and they replenish energy to each node in the form of magnetic coupling resonance. Each MC possesses a maximum battery capacity of *E_MC_*, and its wireless energy radiation range is shown as the fan-shaped region in Figure 2. *R_c_* and *θ* refer to “the farthest reachable charging distance” and “the charging angle of expansion” of the MC, respectively. The values of them are determined by the physical hardware properties of the MC.

In Figure 2, $\vec{CO_{j}}$ indicates the orientation of the current wireless charging radiation region of the *j*-th MC, i.e., the direction of the angle bisector of the fan-shaped area in this figure. $\vec{MC_{j},s_{i}}$ is the direction vector formed between the MC and the node *s_i_* within its charging range. The angle between the above two vectors is denoted as *α_ji_*. Therefore, according to the wireless charging energy receiving model described in [38], the energy receiving power *p^r^*(*s_i_*) of *s_i_* when it is replenished by *MC_j_* is represented as follows.

(1) $$p^{r}\left(s_{i}\right)=\left\{\begin{array}{cc} \mu \frac{\pi \left(\cos\alpha_{ji}+c\right)}{\theta {\left(d\left(MC_{j},s_{i}\right)+\beta \right)}^{2}} & \left(\begin{array}{c} d\in \left(0,R_{c}\right]\\ \alpha \in \left[-\theta /2,\theta /2\right] \end{array}\right)\\ \eta \times p^{c}\left(MC\right) & d==0\\ 0 & d>R_{c} \end{array}\right.$$

In this formula, *d*(*MC_j_*, *s_i_*) represents the distance between *MC_j_* and *s_i_* being recharged by it. The parameters *c*, *μ* and *β* are constants which are determined by the experimental environment and the hardware parameters of chargers. *η* is the energy receiving power coefficient of the node when the MC performs “zero distance-based wireless recharging” on it.

With the bottom left corner of the network as the origin of the rectangular coordinate system and *R_c_* as the radius, the network is divided into several adjacent regular hexagons called “Virtual Grid, VG”, as shown in the dashed range in Figure 1. Obviously, if *MC_j_* stays at the center of a VG, its charging orientation $\vec{CO_{j}}$ only needs to be rotated a maximum of $\left\lceil 2\pi /\theta \right\rceil$ times to achieve wireless energy replenishment for all nodes located within this VG. The VG with its center located inside the network is called “Non-boundary Virtual Grid, NB-VG” (as shown by the regular hexagon with a blue background in Figure 1), and the nodes located inside it are called “Non-boundary nodes, NB nodes”. Similarly, the VG centered outside the network is referred to as the “Boundary Virtual Grid, B-VG” (as shown by the regular hexagon with a yellow background in Figure 1), and the nodes located within the B-VG are referred to as “Boundary nodes, B-nodes”.

According to their task queues, the MCs are enabled to replenish energy to nodes as required. The execution process is described as follows.

**Figure 3:**
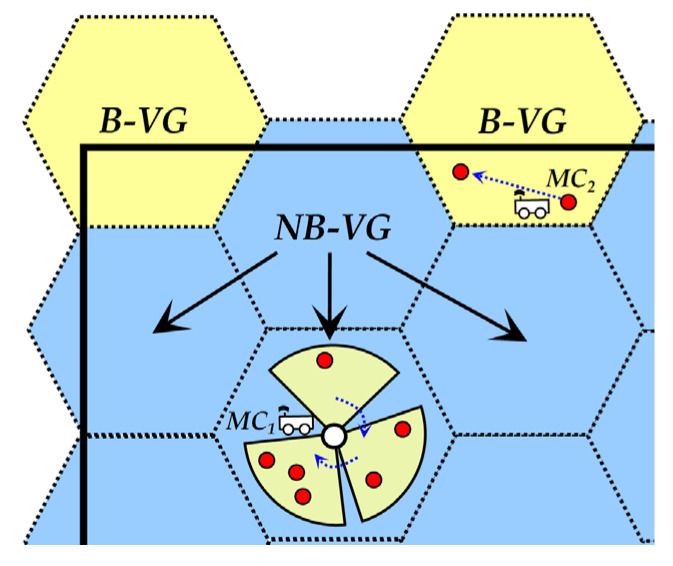
Two charging modes in FLDC.

- For nodes located in NB-VG (i.e., NB-node), the MC adopts a “move-stay-rotate-move” approach to supply energy to the corresponding nodes with a maximum wireless charging radius of *R_c_*. That is, when *MC_j_* reaches a staying point of a NB-VG (the initial location of the staying point is the geometric center of each NB-VG, which may be optimized in the future, as detailed in Section 5.3), it provides energy to the corresponding nodes through multiple rotations of $\vec{CO_{j}}$ according to their distribution, as depicted by *MC*_1_ in Figure 3. Subsequently, *MC_j_* moves to the next staying point.
- For nodes in B-VG (i.e., B-node), the MC will perform a “zero distance-based wireless charging” strategy in a “one-to-one” way, and it leaves this B-VG only after serving all the nodes that need to be replenished in it, as shown by *MC*_2_ in Figure 3.

These two different energy replenishment strategies are adopted in FLDC due to the following two reasons.

- Most of the areas of the NB-VG are within the network; that is to say, there are many nodes located in each of these NB-VGs. These nodes are distributed in some circles whose centers are the geometric centers of the NB-VGs, and the radius of these circles is *R_c_*. Thus, after the MC has continuously radiated energy in different directions at a fixed staying point for a period of time, the energy requirement of all the corresponding nodes in this NB-VG can be satisfied. Obviously, this “one-to-many” wireless charging mode can effectively shorten the movement distance of the MC and ensure its high energy efficiency to a certain extent. In addition, the most suitable radiation direction of the MC at each staying point (the area with the yellow background shown in Figure 3) can be obtained by the discretization method (please see the description in Section 5.4 for details), so that inefficient and ineffective charging behavior can be avoided as much as possible, thereby further improving service efficiency.
- There are only a few nodes in the area of B-VG. Thus, “zero distance-based one-to-one wireless charging” is adopted, which can minimize energy loss without increasing the movement distance of the MC too much. Furthermore, the amount of data generated by these B-nodes located at the edge of the network is significantly less than that of NB-nodes (especially those near the base station). Thus, for the former, the frequency of being recharged is relatively low, which weakens the negative impact of this charging mode to some extent.

It should be noted that the proposed FLDC method can be applied to networks of any shape and scale due to the fact that the network is divided into “virtual hexagons” (hereinafter referred to as “grids”) that is consistent with the omnidirectional energy radiation range of the MC. For convenience of description, we only use a square network as an example to illustrate. Definitions of parameters of the network model are shown in Table 2.

## 4. Parameter Settings and Problem Description

### 4.1. Number of MCs in the Network

In order to adapt to networks with different scales, multiple MCs are allowed to supply energy to each node in a distributed manner in FLDC. Therefore, the value of *N_MC_* needs to be determined at first. According to the description in Section 3, the upper limit value of the energy consumed by an MC for charging all nodes in an NB-VG_k_ (denoted as *E^c^*(*NB-VG_k_*)) can be expressed as follows. Without loss of generality, it is assumed that the energy of each node is recharged from zero to *E_s_*.

(2) $$\begin{array}{l} E^{c}\left(\text{NB-V}G_{k}\right)=\sum_{i=1}^{NoN\left(\mathrm{NB-V}G_{k}\right)}\left(E_{s}\times \frac{p^{c}\left(MC\right)}{p^{r}\left(s_{i}\right)}\right)\\ \;\;\;\;\;=\sum_{i=1}^{NoN\left(\mathrm{NB-V}G_{k}\right)}\left(E_{s}\times \frac{p^{c}\left(MC\right)\times \theta {\left(d\left(MC,s_{i}\right)+\beta \right)}^{2}}{\mu \pi \left(\cos\left(\theta /2\right)+c\right)}\right) \end{array}$$

In Formula (2), *NoN*(*NB-VG_k_*) represents the number of nodes in this non-boundary grid, and *p^c^*(*MC*) is the energy transmission power (i.e., the charging power) of the MC. It should be pointed out that “cos(*θ/*2)” in the denominator of Formula (2) is the minimum value of cos*α_ji_* in Formula (1). In this case, *s_i_* is exactly located on the radius of the fan-shaped energy radiation range of the MC. Thus, the maximum energy consumed by an MC to charge all NB-nodes in the network during a round of service time (denoted as *E^c^_max_*(*NB-VG*)) can be expressed as follows.

(3) $$E_{\max}^{c}\left(\text{NB-VG}\right)=\sum_{k=1}^{Num\left(N\text{B-VG}\right)}E^{c}\left(\text{NB-V}G_{k}\right)$$

In Formula (3), *Num*(*NB-VG*) is the number of NB-VGs which have nodes in need of recharging.

Similarly, the energy *E^c^*(*B-VG_k_*) consumed by an MC to charge all nodes in a B-VG_k_ from zero to full energy *E_s_* can be expressed as follows.

(4) $$E^{c}\left(\text{B-V}G_{k}\right)=\sum_{i=1}^{NoN\left(\text{B-V}G_{k}\right)}\left(E_{s}\times \frac{p^{c}\left(MC\right)}{p^{r}\left(s_{i}\right)}\right)=NoN\left(\text{B-V}G_{k}\right)\times E_{s}\times \eta$$

Here, *NoN*(*B-VG_k_*) is the number of nodes in the boundary grid B-VG_k_. Hence, the maximum energy consumed by an MC to charge all B-nodes in the network during a round of service time (denoted as *E^c^_max_*(*B-VG*)) can be expressed by Formula (5). Similarly, *Num*(*B-VG*) here is the number of B-VGs which have nodes in need of recharging.

(5) $$E_{\max}^{c}\left(\text{B-VG}\right)=\sum_{k=1}^{Num\left(\text{B-VG}\right)}E^{c}\left(\text{B-V}G_{k}\right)$$

Furthermore, during a round of charging, the energy consumed by the MC on moving, i.e., *E^m^*(*NB-VG*), is shown as follows. Here, $\bar{d_{VG}}$ represents the average distance that the MC moves between two adjacent grids (the base station is also regarded as the center of a grid), and *e_m_* is the energy consumption of the MC on moving per unit of distance.

(6) $$E^{\;m}\left(\text{NB-VG}\right)=\bar{d_{VG}}\times e_{m}\times \left(Num\left(\text{NB-VG}\right)+Num\left(\text{B-VG}\right)+1\right)$$

The energy consumption of an MC moving within all the B-VGs which have nodes in need of recharging can be expressed by Formula (7).

(7) $$E^{\;m}\left(\text{B-VG}\right)=\sum_{k=1}^{Num\left(\text{B-VG}\right)}\left(\sum_{i=1}^{NoN\left(\text{B-V}G_{k}\right)-1}e_{m}\times d_{\text{B-VG}}\left(i,\;i+1\right)\right)$$

In addition,

(8) $$\sum_{i=1}^{NoN\left(\text{B-V}G_{k}\right)-1}e_{m}\times d_{\text{B-VG}}\left(i,\;i+1\right)=0\;\;if\;\;NoN\left(\text{B-V}G_{k}\right)==1$$

*d_B-VG_*(*i*, *i *+ 1) is the distance between two adjacent B-nodes (i.e., *s_i_* and *s_i+_*_1_) that are sequentially traversed by the MC.

In summary, if inequality (9) holds, it can ensure that the charging demand of all nodes in the network can be met. *E_MC_* here is the maximum amount of energy that an MC can carry.

(9) $$E_{\max}^{c}\left(\text{NB-VG}\right)+E_{\max}^{c}\left(\text{B-VG}\right)+E^{\;m}\left(\text{NB-VG}\right)+E^{\;m}\left(\text{B-VG}\right)\leq N_{MC}\times E_{MC}$$

Thus, the number of MCs needed in the network can be calculated out by Formula (10).

(10) $$N_{MC}=\left\lceil \frac{E_{\max}^{c}\left(\text{NB-VG}\right)+E_{\max}^{c}\left(\text{B-VG}\right)+E^{\;m}\left(\text{NB-VG}\right)+E^{\;m}\left(\text{B-VG}\right)}{E_{MC}}\right\rceil$$

### 4.2. Maximum Duration T_max_ for a Single MC to Finish a Round of Service

As mentioned above, in FLDC, the MCs periodically recharge each node. Due to the limited amount of energy carried by each MC, we first discuss the maximum duration of a round of energy replenishment.

When assigning the charging task to *N_MC_* MCs, it can be considered that the number of NB-VG and B-VG that each MC can serve in a round of traversal time is *Num*(*NB-VG*)/*N_MC_* and *Num*(*B-VG*)/*N_MC_*, respectively. Therefore, the upper limit values of the duration for an MC to charge all nodes in NB-VG and B-VG that it can serve (denoted as *T^c^_max_*(*NB-VG*) and *T^c^_max_*(*B-VG*), respectively) can be expressed as follows.

(11) $$T_{\max}^{c}\left(\text{NB-VG}\right)=E_{s}\times \frac{\theta {\left(\bar{d\left(MC_{j},s_{i}\right)}+\beta \right)}^{2}}{\mu \pi \left(\cos\left(\theta /2\right)+c\right)}\times \min\left(\left\lceil \frac{2\pi }{\theta }\right\rceil ,\;\bar{n_{\text{NB-VG}}}\right)\times \frac{Num\left(\text{NB-VG}\right)}{N_{MC}}$$

(12) $$T_{\max}^{c}\left(\text{B-VG}\right)=\frac{E_{s}}{N_{MC}}\times \frac{1}{\eta \times p^{c}\left(MC\right)}\times \sum_{k=1}^{Num\left(\text{B-VG}\right)}NoN\left(\text{B-V}G_{k}\right)$$

In Formula (11), $\bar{d\left(MC_{j},s_{i}\right)}$ represents the mean distance between all NB-nodes and the center of the NB-VG they are located in (i.e., the staying point of *MC_j_* in this NB-VG). It should be pointed out that if the average number of nodes in each NB-VG (denoted as $\bar{n_{\text{NB-VG}}}$) is less than the maximum number of directions that the MC can charge at each staying point (marked as $\left\lceil 2\pi /\theta \right\rceil$), it indicates that there are no nodes in the area covered by certain charging directions of the MC. Obviously, in this case, the MC only needs to perform a period of $\bar{n_{\text{NB-VG}}}$ charging at these staying points at most. Otherwise, a period of $\left\lceil 2\pi /\theta \right\rceil$ energy replenishment process should be carried out. The value of $\bar{n_{\text{NB-VG}}}$ is calculated by Formula (13).

(13) $$\bar{n_{\text{NB-VG}}}=\sum_{k=1}^{Num\left(\text{NB-VG}\right)}NoN\left({\text{NB-VG}}_{k}\right)/Num\left(\text{NB-VG}\right)$$

Moreover, Formula (14) shows the expression of the time spent on moving during a round of energy replenishment service, in which *v* is the speed of the MC.

(14) $$T^{m}=\frac{\left(\begin{array}{l} \bar{d_{VG}}\times \left(Num\left(\text{NB-VG}\right)+Num\left(\text{B-VG}\right)+1\right)\\ +\sum_{k=1}^{Num\left(\text{B-VG}\right)}\left(\sum_{i=1}^{NoN\left(\text{B-V}G_{k}\right)-1}d_{\text{B-VG}}\left(i,\;i+1\right)\right) \end{array}\right)}{N_{MC}\times v}$$

And,

(15) $$\sum_{i=1}^{NoN\left(\text{B-V}G_{k}\right)-1}d_{\text{B-VG}}\left(i,\;i+1\right)=0\;\;\;if\;\;NoN\left(\text{B-V}G_{k}\right)==1$$

Therefore, the periodic recharging process of each MC can be carried out smoothly if and only if Formula (16) holds.

(16) $$T_{\max}\leq T_{\max}^{c}\left(\text{NB-VG}\right)+T_{\max}^{c}\left(\text{B-VG}\right)+T^{m}$$

### 4.3. Problem Description

The objective of FLDC is to design a flexible and adaptive dynamic charging scheduling scheme for the network model mentioned above as well as the energy replenishment plan of the MC in traversing each NB-VG and B-VG, so as to maximize the efficiency of all nodes in the network. Hence, the charging benefit of node *s_i_* (denoted as *U^c^*(*s_i_*)) is defined as follows.

(17) $$U^{c}\left(s_{i}\right)=\frac{\varepsilon \times \mu \left(s_{i}\right)}{\varepsilon \times \mu \left(s_{i}\right)+\left(1-\varepsilon \right)\times \sigma \left(s_{i}\right)}$$

1. In Equation (17), *μ*(*s_i_*) represents the proportion of “the extended lifetime of *s_i_* after being recharged once” to “the maximum lifetime it can survive without being recharged”. That is,
  (18) $$\mu \left(s_{i}\right)=\frac{\left(E_{s}/p\left(s_{i}\right)\right)-t^{r}\left(s_{i}\right)}{\left(E_{s}/p\left(s_{i}\right)\right)}$$
  p(si) is the energy consumption rate of si, and tr(si) represents its residual lifetime at the moment before the MC starts to charge it. Namely,
  (19) $$t^{r}\left(s_{i}\right)=\left(E_{s}-t^{w}\left(s_{i}\right)\times p\left(s_{i}\right)\right)/p\left(s_{i}\right)$$
  In (19), tw(si) represents the active duration of si from the last time it has been recharged to the current moment.
2. The definition of *σ*(*s_i_*) in Formula (17) is shown as follows.
  (20) $$\sigma \left(s_{i}\right)=\left.\frac{t^{c}\left(s_{i}\right)-\min\left(t^{c}\left(s_{j}\right)\right)}{\max\left(t^{c}\left(s_{j}\right)\right)-\min\left(t^{c}\left(s_{j}\right)\right)}\;\right|\;j\in \left[1,N\right]$$
  And,
  (21) $$t^{c}\left(s_{i}\right)=\left(E_{s}-t^{r}\left(s_{i}\right)\times p\left(s_{i}\right)\right)/p^{r}\left(s_{j}\right)$$
  max(*t^c^*(*s_j_*)|*j*∈[1, *N*]) and min(*t^c^*(*s_j_*)|*j*∈[1, *N*]) represent the longest and the shortest charging time of all nodes being recharged, respectively. It is easy to see that when the following three conditions are met simultaneously, the time spent on charging *s_j_* is the longest.
  - The distance between *s_j_* and the staying point is exactly *R_c_*;
  - *s_j_* is located at the edge of the fan-shaped energy radiation range of the MC;
  - *s_j_* is recharged from no energy to its full energy capacity, *E_s_*. That is,
  (22) $$\max\left(t^{c}\left(s_{j}\right)\right)=\left(\frac{E_{s}\times \theta {\left(R_{c}+\beta \right)}^{2}}{\mu \pi \left(\cos\left(\theta /2\right)+c\right)}\right)/p^{r}\left(s_{j}\right)$$
  Without loss of generality, we assume that the amount of energy supplied to any node *s_j_* should no less than 2*T_max_* × *p*(*s_j_*) every time, which means that the extended lifetime of *s_j_* after being recharged must be at least equal to the longest time interval between the two services provided by the MC to it. Thus, the following equation holds.
  (23) $$\min\left(t^{c}\left(s_{j}\right)\right)=\left(2T_{\max}\times p\left(s_{j}\right)\right)/p^{r}\left(s_{j}\right)$$
  It is not difficult to find that σ(si) reflects the approximation degree between the duration of one charge of si and the longest charging duration among all nodes during this round.
3. In (17), *ε* is an adjustment coefficient, with a value between 0 and 1. When its value is close to 1, the extended lifetime of *s_i_* after being recharged is the main indicator to show its charging efficiency. That is, the larger the value, the higher the charging efficiency of *s_i_*. On the contrary, when *ε* is close to zero, the duration of a single charge for *s_i_* has a greater impact on its charging efficiency. That is, the smaller the value, the higher the charging efficiency of *s_i_*.

At this point, the objective and constraints of FLDC can be described as follows. Definitions of parameters in this section are shown in Table 3.

$$\max\left(\sum_{i=1}^{N}U^{c}\left(s_{i}\right)\right)\;s.t.\;E_{\max}^{c}\left(\text{NB-VG}\right)+E_{\max}^{c}\left(\text{B-VG}\right)+E^{\;m}\left(\text{NB-VG}\right)+E^{\;m}\left(\text{B-VG}\right)\leq N_{MC}\times E_{MC}\;T_{\max}\leq T_{\max}^{c}\left(\text{NB-VG}\right)+T_{\max}^{c}\left(\text{B-VG}\right)+T^{m}$$

## 5. Method Description

To maximize the charging efficiency of nodes in a “one-to-many wireless energy supply mode with rotatable charging direction”, the following three tasks are carried out by FLDC. Firstly, with the help of a two-layer fuzzy logic system, the priority of each grid can be obtained, which reasonably determines the order in which the MC traverses each staying point, ensuring the fairness of nodes being recharged. Subsequently, the gradient descent method is adopted to optimize the position of each staying point, which shortens the average distance between nodes in need of recharging and the MC, effectively reducing energy loss during the charging process. Finally, based on the priority level of nodes being replenished, the discrete energy radiation directions (charging direction angles) as well as the rotation order of the MC at each staying point are determined in sequence. Furthermore, with the goal of balancing the energy receiving efficiency of each node, fine adjustment of charging directions is made to shorten the overall charging time.

### 5.1. Priority of Node and Grid Based on Two-Layer Fuzzy Logic

In FLDC, the attributes of *s_i_* at any time can be characterized by its residual lifetime *t^r^*(*s_i_*), energy consumption rate *p*(*s_i_*), and the distance from the center of the grid (marked as *d*(*s_i_*, *center_k_*)) it belongs to. Obviously, nodes with a smaller value of *t^r^*(*s_i_*) or a larger value of *p*(*s_i_*) should be prioritized for energy replenishment as much as possible. In addition, the distance between the center of a grid and the current position of the MC (denoted as *d*(*center_k_*, *MC_j_*)) also has a significant impact on its traversal order. To address this, we construct a two-layer fuzzy logic system using the aforementioned attributes to determine a reasonable node-charging priority, and subsequently the traversal order of each grid.

Fuzzy logic is a method of decision-making that utilizes human thinking and reasoning. The system calculates fuzzy inputs and provides outputs ranging from 0 to 1 [39]. In this section, we use *t^r^*(*s_i_*), *p*(*s_i_*) and *d*(*s_i_*, *center_k_*) as inputs to the first layer of the fuzzy logic system to obtain the Node Priority (NP) of nodes being charged in each grid. Then, NP and *d*(*center_k_*, *MC_j_*) are regarded as the inputs to the second layer of this system to obtain the Grid Priority (GP) of each grid being traversed.

The trapezoidal and triangular membership functions are adopted in the proposed fuzzy logic system. Namely,

(24) $$Trapezoida\;l(x)=\left\{\begin{array}{cc} 0 & x\leq x_{1}\\ \left(x-x_{1}\right)/\left(x_{2}-x_{1}\right) & x_{1}<x\leq x_{2}\\ 1 & x_{2}<x\leq x_{3}\\ \left(x_{4}-x\right)/\left(x_{4}-x_{3}\right) & x_{3}<x\leq x_{4}\\ 0 & x>x_{4} \end{array}\right.$$

(25) $$Triangular\left(x\right)=\left\{\begin{array}{cc} 0 & x\leq x_{1}\\ \left(x-x_{1}\right)/\left(x_{2}-x_{1}\right) & x_{1}<x\leq x_{2}\\ \left(x_{3}-x\right)/\left(x_{3}-x_{2}\right) & x_{2}<x\leq x_{3}\\ 0 & x>x_{3} \end{array}\right.$$

Here, *x* is the clear input, and *x*_1_–*x_4_* is the coordinate range of the membership function of the fuzzy variable.

The three types of input language values and membership function ranges in the first layer of the system are shown in Table 4, and the system output is shown in Table 5. Here, max(*p*(*s_i_*)) represents the node with the highest energy consumption rate among all nodes participating in the calculation. In addition, the value of *d*(*s_i_*, *center_k_*) clearly does not exceed *R_c_*. Similarly, the value of *t^r^*(*s_i_*) will not exceed 2*T* according to the trigger conditions of the MC mentioned in Section 5.2.

**Figure 4:**
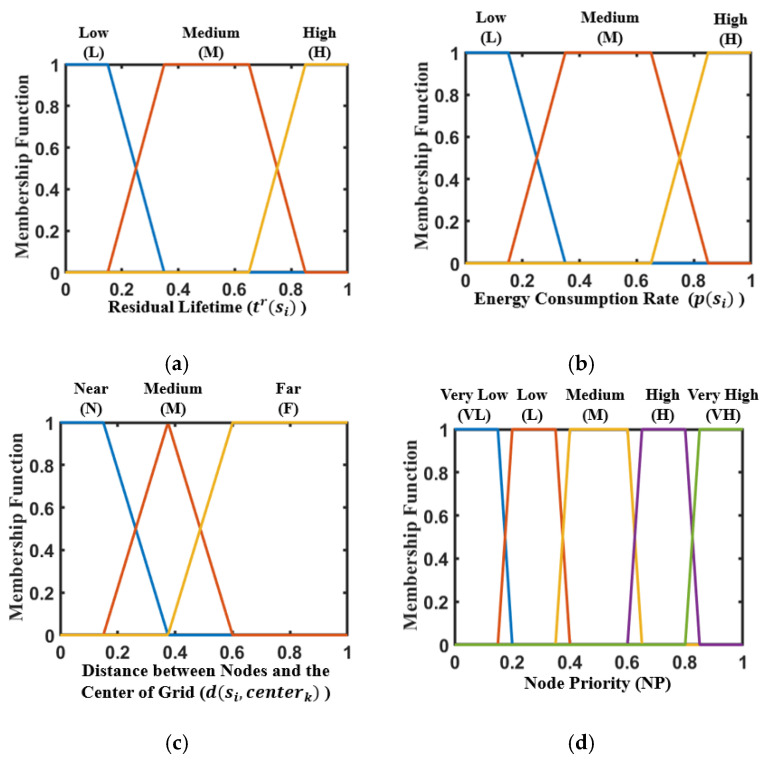
Input and output function of the first layer. (**a**) Residual lifetime; (**b**) energy consumption rate; (**c**) distance between nodes and the center of grid; (**d**) node priority.

The input-output functions of the fuzzy logic system in this layer are shown in Figure 4. It can be seen that by transforming the clear inputs into fuzzy inputs through membership functions, three types of membership degrees of nodes regarding this attribute can be obtained. Then, the fuzzy inputs can be transformed into fuzzy outputs according to the IF-THEN rule. The first layer of the fuzzy logic system has three types of inputs and one type of output, with a total of twenty-seven rules, as shown in Table 6. Without loss of generality, nodes with lower residual lifetimes and higher energy consumption rates will have higher priority, as the output of rule R7 shows. On the contrary, the priority of nodes with higher residual lifetimes and lower energy consumption rates is relatively lower, as the output of rule R19 shows.

The fuzzy output of the above two membership functions are calculated as follows.

(26) $$\mu_{Trapezoidal}=\max\left(\min\left(\frac{x-x_{1}}{x_{2}-x_{1}},\;1,\;\frac{x_{4}-x}{x_{4}-x_{3}}\right),\;0\right)$$

(27) $$\mu_{Triangular}=\max\left(\min\left(\frac{x-x_{1}}{x_{2}-x_{1}},\;\frac{x_{3}-x}{x_{3}-x_{2}}\right),\;0\right)$$

With the help of the fuzzy input function, three fuzzy output membership degrees can be calculated out. By taking the minimum value for each rule mentioned above, the corresponding fuzzy output for each rule can be obtained. Then, we take the maximum linguistic value for each output to obtain the modified membership function image for each node.

Subsequently, the centroid-based deblurring method is adopted to obtain the deblurring points. The fuzzy output is transformed into a clear one, and then different expected values are sampled and averaged. The sum of the contributions of each sample point to the overall membership degree is calculated as the current priority. Assuming that *μ*(*Z_p_*) is the modified membership function of the fuzzy output set, and *Num* is the number of points on the modified output that extends along the *x*-axis, then

(28) $$COG=\sum_{p=1}^{Num}Z_{p}\mu \left(Z_{p}\right)/\sum_{p=1}^{Num}\mu \left(Z_{p}\right)$$

In this way, the priority of each node in the current set of nodes to be recharged can be obtained in a range from zero to one. The larger the value, the higher the priority. Without loss of generality, the highest NP value of all nodes in each grid is considered as the unified NP value of nodes in that grid and is used as an input into the second layer of the fuzzy logic system. In addition, the distance between the center of each grid and the current position of the MC (*d*(*center_k_*, *MC_j_*)) is also input. From this, the input-output rules and membership functions of the second layer can be obtained, as shown in Table 7 and Table 8. Here, max(*NP*(*s_i_*)) represents the highest value of NP among all nodes participating in the calculation, while max(*d*(*center_k_*, *MC_j_*)) is the maximum distance between the center of each grid and the current position of the MC.

**Figure 5:**
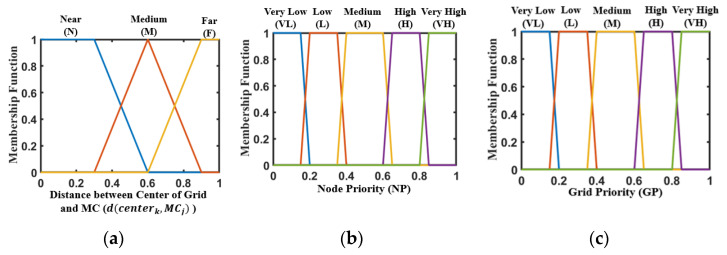
Input and output function of the second layer. (**a**) Distance between center of grid and MC; (**b**) node priority; (**c**) grid priority.

The input and output functions of the fuzzy logic system in this layer are shown in Figure 5.

The calculation rules for the second layer of fuzzy logic are shown in Table 9. Obviously, if the grid is closer to the current position of the MC and the value of its *NP*(*s_i_*) is high at this time, it will obtain a higher priority of being traversed in the future, as shown in rule R5. On the contrary, if the grid is far away from the MC and its *NP*(*s_i_*) is low, its traversal order will be later, as rule R11 shows.

### 5.2. Grid Priority-Based Charging Process of the MC

According to the description in Section 5.1, the priority of each grid being traversed at any time can be obtained with the help of the double-layer fuzzy logic system. Thus, the charging process of the MC in FLDC is described as follows.

Step 1. When the network has been deployed, the base station continuously monitors the status of all nodes. If the residual lifetime of any node (i.e., *t^r^*(*s_i_*)) decreases to *T_max_*, IDs of nodes whose *t^r^*(*s_i_*) is less than 2*T_max_* are put into the “set of nodes to be recharged”. This set is denoted as *Q_node_*, and it is initially empty. Afterwards, the BS will perform the above operation once every *T_max_* interval. As described in Section 4.2, *T_max_* is defined as the maximum duration required for a single MC to complete a round of energy replenishment. That is to say, for any node that MC is about to serve in this round, as long as it can survive for at least *T_max_* time from the departure time of the MC, it can ensure that it will not die and can be successfully replenished before the MC arrives. Furthermore, it is not difficult to see that nodes with a lifetime of less than 2*T_max_* at this moment probably need to be recharged during the next two rounds of MC traversal (otherwise they may die). Therefore, the above settings are selected.

Step 2. The priority of each grid corresponding to the nodes in *Q_node_* are calculated with the help of the two-layer fuzzy logic system.

Step 3. Each MC located at the base station selects a grid with the highest priority in sequence as its target to serve, and the ID of each node in it to be recharged is deleted from *Q_node_*. Then, *MC_j_* moves from the BS to the corresponding grid. If the target of *MC_j_* is an NB-VG, it just moves to its staying point. If it is a B-VG, *MC_j_* needs to move to the node with the highest value of NP in it.

Step 4. After arriving at the grid, *MC_j_* will serve all the nodes which are located in it and waiting to be recharged according to the method described in Section 3. That is, if its charging objects are NB-nodes, *MC_j_* rotates its charging orientation in a discrete way to achieve a “one-to-more” charging. Otherwise, the “zero distance-based one-to-one wireless recharging” mode will be adopted.

Step 5. If *Q_node_* = Ø when *MC_j_* completes serving all the nodes in a grid (marked as *VG_k_*), it indicates that there are no nodes waiting to be recharged in the network at this time. In this case, it returns to the base station, and the algorithm jumps to step 6 for execution. Otherwise, it selects the grid with the highest priority (marked as *VG_k+_*_1_) corresponding to the nodes in *Q_node_* at this time. Subsequently, *MC_j_* judges whether it can continue to traverse the next grid according to (29) and (30). If both inequalities hold true, *MC_j_* deletes the IDs of nodes to be recharged in *VG_k+_*_1_ from *Q_node_*, and then moves to this grid to serve these nodes. After completing these tasks, the judgment of this step will be carried out again until it returns back to the base station.

(29) $$E^{r}\left(MC_{j}\right)\geq E^{c}\left(VG_{k+1}\right)+e_{m}\times d\left(k,\;k+1\right)+e_{m}\times d\left(k+1,\;BS\right)$$

(30) $$T_{\max}-T\left(MC_{j}\right)\geq T^{c}\left(VG_{k+1}\right)+\frac{d\left(k,\;k+1\right)+d\left(k+1,\;BS\right)}{v}$$

*E^r^*(*MC_j_*) and *T*(*MC_j_*) are the residual energy of *MC_j_* and the duration during which it has served in this round, respectively. *d*(*k*, *k *+ 1) and *d*(*k *+ 1, BS) represent the distance between the *k*-th grid (current grid) and the (*k *+ 1)-th grid (the next grid *MC_j_* intends to traverse), as well as the distance between the (*k *+ 1)-th grid and the base station, respectively. Moreover, the energy and time that *MC_j_* is expected to consume in serving the (*k *+ 1)-th grid are marked as *E^c^*(*VG_k+_*_1_) and *T^c^*(*VG_k+_*_1_), respectively. Thus, the following two conclusions can be drawn.

- If the grid is an NB-VG, the value of *E^c^*(*VG_k+_*_1_) can be estimated by Formula (2). Moreover, the value of *T^c^*(*VG_k+_*_1_) in Formula (30) can be expressed by (31), in which *N^c^*(*NB-VG_k+_*_1_) represents the number of nodes waiting to be recharged in the grid.
  (31) $$T^{c}\left(VG_{k+1}\right)=E_{s}\times \frac{\theta {\left(\bar{d\left(MC_{j},s_{i}\right)}+\beta \right)}^{2}}{\mu \pi \left(\cos\left(\theta /2\right)+c\right)}\times \min\left(\left\lceil \frac{2\pi }{\theta }\right\rceil ,\;N^{c}\left(\text{NB-V}G_{k+1}\right)\right)$$
- If the grid is B-VG, Formula (4) is used to estimate the value of *E^c^*(*VG_k+_*_1_). In this case, the value of *T^c^*(*VG_k+_*_1_) in (30) should be calculated using Formula (32). Here, the number of nodes waiting to be recharged in the grid is denoted as *NoN^c^*(*B-VG_k+_*_1_).
  (32) $$T^{c}\left(VG_{k+1}\right)=E_{s}\times \frac{1}{\eta \times p^{c}\left(MC\right)}\times NoN^{c}\left(\text{B-V}G_{k+1}\right)$$

Step 6. When *MC_j_* returns back to the BS, its battery will be replaced. If *Q_node_* ≠ Ø at this moment, the grid with the highest priority corresponding to the nodes in *Q_node_* is selected with the help of the two-layer fuzzy logic system again. Then, the algorithm jumps to step 3 to start a new round of charging service. Otherwise, *MC_j_* stays at the BS until *Q_node_* is not empty again, and then the above steps are carried out once more.

### 5.3. Optimization of the Location of Staying Points (OLSP) for Minimizing the Sum of Charging Distance

**Figure 6:**
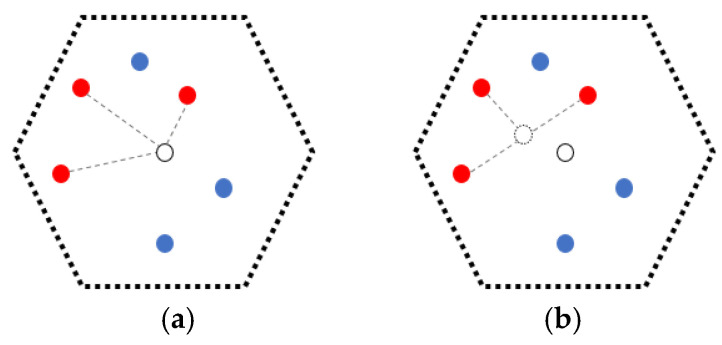
Location of the staying point before and after optimization. (**a**) the staying point is the geometric center of NB-VG_k_. (**b**) the staying point is the centroid of Δ*s_i_s_j_s_k_*.

As mentioned above, the staying points of the MC in each NB-VG are initially set at their geometric centers. However, due to the random distribution of nodes in the grid, this position may not be the “best staying point”. For example, in Figure 6, there are only three nodes (*s_i_*, *s_j_*, *s_k_*) waiting to be recharged in NB-VG_k_. Obviously, if the staying point of the MC is set to the centroid of Δ*s_i_s_j_s_k_*, the charging effect is inevitably better than that being set to the geometric center *P* of NB-VG_k_. The reason is that the energy receiving power *p^r^*(*s_i_*) is almost inversely proportional to the value of *d*(*MC_j_*, *s_i_*) as a square term according to Formula (1). In addition, as described in Section 5.2, the IDs in *Q_node_* often change, which means that even for the same grid, the nodes served by the MC may not be exactly the same each time.

In summary, it is necessary to optimize the location of each staying point traversed by the MC in each round of the recharging process. The specific steps are described as follows.

Step 1. When the MC is about to traverse grid NB-VG_k_, the following function describing the sum of distances between the staying point *SP_k_* and nodes located within it and waiting to be recharged can be obtained. The coordinates of *SP_k_* and any node *s_i_* are denoted as (*x_k_*, *y_k_*) and (*x*(*s_i_*), *y*(*s_i_*)), respectively, and the former is initially the geometric center of NB-VG_k_.

(33) $$D\left(N\text{B-V}G_{k}\right)=\sum_{i=1}^{N^{c}\left(N\text{B-V}G_{k}\right)}\sqrt{{\left(x\left(s_{i}\right)-x_{k}\right)}^{2}+{\left(y\left(s_{i}\right)-y_{k}\right)}^{2}}$$

Step 2. The gradient of *D*(*NB-VG_k_*) relative to the location of the current staying point (*x_k_*, *y_k_*) is calculated, as shown in Formula (34).

(34) $$\nabla D\left(N\text{B-V}G_{k}\right)=\partial D\left(\cdot \right)/x_{k}+\partial D\left(\cdot \right)/y_{k}$$

Obviously, the value of *D*(*NB-VG_k_*) can be minimized by adjusting the position of this staying point in the opposite direction of the gradient.

Step 3. The possible updated coordinate (*x_k_*^(*j*+1)^, *y_k_*^(*j*+1)^) of the staying point in the grid is calculated using Formula (35). Here, *j* represents the number of times this position has been updated, and (*x_k_*^(0)^, *y_k_*^(0)^) is just (*x_k_*, *y_k_*). Moreover, *η* represents the step size for each movement during this adjustment process, and *D*(*NB-VG_k_*)^(*j*+1)^ represents the result of the *j*-th calculation of gradient.

(35) $$\left(x_{k}^{\left(j+1\right)},\;y_{k}^{\left(j+1\right)}\right)=\left(x_{k}^{\left(j\right)},\;y_{k}^{\left(j\right)}\right)-\eta \nabla D{\left(\text{NB-V}G_{k}\right)}^{\left(j+1\right)}$$

Step 4. We judge whether the following three conditions are met if the location of the staying point is updated to (*x_k_*^(*j*+1)^, *y_k_*^(*j*+1)^). If so, the staying point of this grid is moved along the direction of the gradient mentioned above to the updated coordinate calculated in Formula (35). Next, the value of *j* is updated to *j *+ 1, and then the algorithm jumps to the third step to continue optimizing the location of the staying point. Otherwise, (*x_k_*^(*j*+1)^, *y_k_*^(*j*+1)^) is taken as the final result of the position of this staying point.

- The first condition. For any node waiting to be recharged in NB-VG_k_, the distance between it and the updated staying point does not exceed the upper limit value of the wireless charging distance, i.e., *R_c_*. That is to say, inequality (36) holds.
  (36) $$\left.\sqrt{{\left(x\left(s_{i}\right)-x_{k}^{\left(j\right)}\right)}^{2}+{\left(y\left(s_{i}\right)-y_{k}^{\left(j\right)}\right)}^{2}}\leq R_{c}\;\right|\;s_{i}\in Q_{node}$$
- The second condition. The distance between the two staying points after the *j*-th and (*j*−1)-th adjustments is greater than the threshold *δ*, meaning that Formula (37) is valid.
  (37) $$\sqrt{{\left(x_{k}^{\left(j\right)}-x_{k}^{\left(j-1\right)}\right)}^{2}+{\left(y_{k}^{\left(j\right)}-y_{k}^{\left(j-1\right)}\right)}^{2}}>\delta$$
- The third condition. The number of adjustments *j* has not reached the upper limit.

### 5.4. Determining the Charging Directions Based on the Discretization Method

After the OLSP has been executed, the location of the staying point in each grid should be as close as possible to the nodes waiting to be recharged. However, according to the output mechanism of the first layer of the fuzzy logic system described in Section 5.1, even within the same grid, the priority of each node being recharged often varies. In addition, we need to further determine the direction of energy radiation as well as the rotation sequence of the MC’s charging orientation. Therefore, the process of “Determining the Charging Directions” (DCD) is described as follows.

Step 1. Nodes whose IDs are in *Q_node_* are sorted in a descending order of their NP in NB-VG_k_ to form a queue *Q*(*NB-VG_k_*). Obviously, the length of this queue is *N^c^*(*NB-VG_k_*).

Step 2. A polar coordinate system is established with (*x_k_*, *y_k_*) as the pole. Thus, the polar coordinates of those *N^c^*(*NB-VG_k_*) nodes mentioned above can be calculated. That is, for any *s_i_* pending recharge in NB-VG_k_, its polar coordinate can be expressed as (*d*(*SP_k_*, *s_i_*), *φ_i_*).

Step 3. The direction vector $\vec{SP_{k}s_{i}}$ from the staying point to the first node in *Q*(*NB-VG_k_*) is taken as the initial orientation of *MC_j_*’s first energy radiation range. Then, IDs of nodes located in this fan-shaped area are put into *C*_1_ which is defined as the set of nodes being covered by *MC_j_* in this orientation. According to Formula (1), it can be seen that in the case where *d*(*MC_j_*, *s_i_*) is fixed, *p^r^*(*s_i_*) can reach its maximum value if and only if *α_ji_* is zero. Therefore, the purpose of this is to ensure that the node with the highest NP in *Q*(*NB-VG_k_*) as well as its neighbor can be efficiently and preferentially recharged.

Step 4. The IDs that were already in *C*_1_ are deleted from *Q*(*NB-VG_k_*). Then, if *Q*(*NB-VG_k_*) is not empty at this time, the algorithm goes to the third step and iteratively executes it to form the next cover set *C*2, *C3*, etc. Otherwise, the execution process of DCD ends, and the discrete charging directions of *MC_j_* in NB-VG_k_ and the respective cover sets can be obtained. It should be pointed out that there may be overlap between those fan-shaped regions, but any node will only be recharged once during *MC_j_*’s residence in NB-VG_k_. That is, *C*_1_∩*C*_2_∩*C*_3_… = *Ø* and |*C*_1_| + |*C*_2_| + |*C*_3_|… = *N^c^*(*NB-VG_k_*).

**Figure 7:**
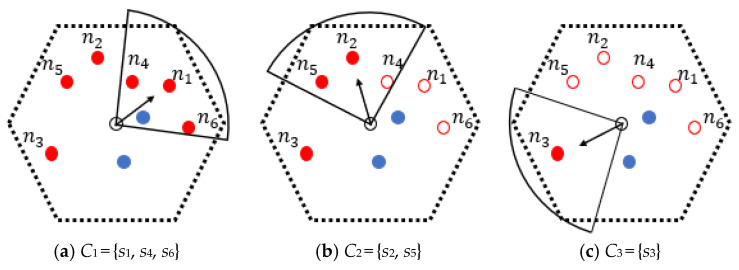
Process of determining the initial charging directions.

An example of the execution process of DCD is described as follows. As shown in Figure 7a, there are eight nodes in NB-VG_k_, and six of them are waiting to be recharged (red dots). The NPs of all these nodes have been calculated. Without loss of generality, we let *Q*(*NB-VG_k_*) = {*s*_1_, *s*_2_, *s*_3_, *s*_4_, *s*_5_, *s*_6_}, and *SP_k_* is the staying point of this grid obtained by the OLSP algorithm.

So, $\vec{SP_{k}s_{1}}$ is regarded as the initial direction vector of the first energy radiation range of *MC_j_*. It can be seen from Figure 7a that both *s_4_* and *s_6_* are located within the fan-shaped charging area corresponding to this orientation. Thus, *C*_1_ = {*s*_1_, *s*_4_, *s*_6_}, and the IDs in *Q*(*NB-VG_k_*) are now updated to {*s*_2_, *s*_3_, *s*_5_}. Subsequently, $\vec{SP_{k}s_{2}}$ is set as the initial direction vector of the second charging range, as shown in Figure 7b, and it is easy to see that *s_5_* is covered by it, that is, *C*_2_ = {*s*_2_, *s*_5_}. Similarly, $\vec{SP_{k}s_{3}}$ is marked as the initial direction vector of the third energy radiation range of *MC_j_*, and now *C_3_* = {*s*_3_}, as shown in Figure 7c.

It can be seen that after carrying out the DCD algorithm, the charging direction vectors all point to the node with the highest value of NP covered by the corresponding energy radiation area. Although this can maximize the value of *p^r^*(*s_i_*), it may affect the effectiveness of other nodes being served. For example, in Figure 7a, due to $∠\vec{SP_{k}s_{1}}\vec{SP_{k}s_{4}}$ being large and the distance between *s_4_* and *SP_k_* being relatively far, the value of *p^r^*(*s*_4_) is lower. To further balance the charging efficiency among nodes in the same set, the charging direction of the MC needs to be fine-tuned, which is named “Determining the Charging Directions after Adjustment” (DCD_A). The final direction vector $\vec{SP_{k}\left(C_{p}\right)}$ for *MC_j_* to charge nodes in the set *C_p_* at *SP_k_* can be expressed by Formula (38).

(38) $$\vec{SP_{k}\left(C_{p}\right)}=\sum_{i=1}^{\left|C_{p}\right|}\left(\frac{{\left(d\left(SP_{k},s_{i}\right)\right)}^{2}}{\sum_{i=1}^{\left|C_{p}\right|}{\left(d\left(SP_{k},s_{i}\right)\right)}^{2}}\times \phi_{i}\right)$$

**Figure 8:**
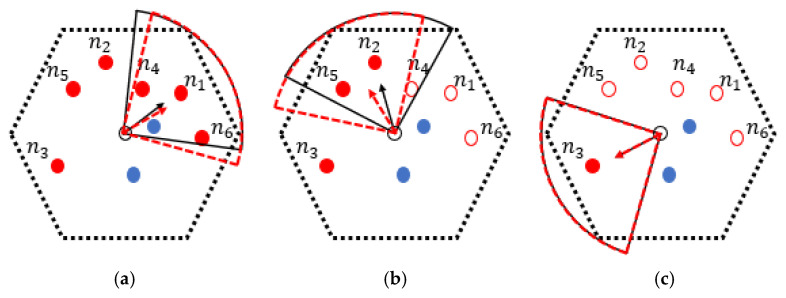
Final charging directions and the radiation ranges of the MC. (**a**) modify the first charging direction of the MC. (**b**) modify the second charging direction of the MC. (**c**) the third charging direction of the MC doesn’t need to be corrected.

${\left(d\left(SP_{k},s_{i}\right)\right)}^{2}/\sum_{i=1}^{\left|C_{p}\right|}{\left(d\left(SP_{k},s_{i}\right)\right)}^{2}$ here is taken as the weight of *φ_i_* of each node in *C_p_* associated with *SP_k_*. That is, the final orientation of the MC when serving nodes in *C_p_* will be more inclined towards the node that is farther away from it. Figure 8 shows the final charging directions as well as the radiation range of the MC located at *SP_k_* after the above corrections. It is not difficult to see from both Figure 8a,b that these charging directions take into account the fairness of node recharge efficiency in the covered area. This shortens the charging and staying duration of the MC, which improves its service efficiency. It should be noted that there is only one node *s_3_* covered by the third energy radiation range (as shown in Figure 8c). Thus, there is no need to modify the charging direction of the MC in this case.

Therefore, when the MC traverses to any NB-VG_k_, it will supply energy to nodes one by one according to the order constructed by the DCD method, with fine-tuned results for its charging directions as mentioned above.

## 6. Simulation Results

In order to verify the performance of FLDC, a series of simulations are carried out to show the charging utility and energy utilization ratio of the MC as well as the charging duration. Three methods, BASE, BASE + OLSP, and BASE + DCD_A are selected as the comparison algorithms.

- BASE is the benchmark method for FLDC in which the MC only stays at the geometric center when traversing each NB-VG (the OLSP is not carried out), and the charging orientation of the MC is not adjusted (no DCD_A is performed).
- BASE + OLSP and BASE + DCD_A are the variations of BASE that include the “optimization of the location of staying point” and “determining the charging directions after adjustment”, respectively.

We also compare FLDC with two typical directional charging scheduling algorithms, AEC and MUC, on a number of dead nodes, energy consumption and movement distance of the MC during a round of charging time, and the node charging utility. Parameter settings of the simulation are shown in Table 10. It should be noted that, for all methods, if not specified, each node served by the MC must be recharged to its full battery capacity, i.e., *Es*.

### 6.1. Impact of the Adjustment Coefficient ε on Charging Utility

**Figure 9:**
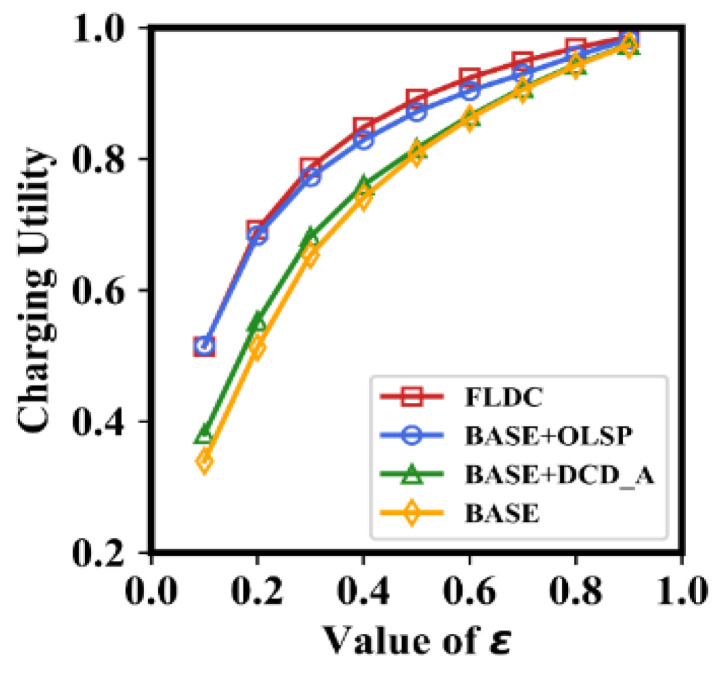
Charging utility under different values of *ε*.

As mentioned above, maximizing the energy replenishment efficiency in directional charging mode is the main purpose of FLDC. Thus, the result of *U^c^*(*s_i_*) for BASE, BASE + OLSP, BASE + DCD_A, and FLDC with different values of *ε* are analyzed first. Without loss of generality, we present the average value of *U^c^*(*s_i_*) of all nodes (marked as $\bar{U^{c}\left(s_{i}\right)}$) in each method in Figure 9.

In Figure 9, the values of $\bar{U^{c}\left(s_{i}\right)}$ for all the four methods increase with increasing *ε*, but the gap between them continues to narrow. According to Formula (17), the larger the *ε*, the higher the proportion of *μ*(*s_i_*) in the fraction of its molecule. For this reason, the value of *U^c^*(*s_i_*) also increases and continuously approaches one. Regardless of the value of *ε*, the charging utility in BASE is the lowest one among the four methods, with the difference being more pronounced when *ε* is small. This is because it does not optimize the MC’s staying point location nor its charging direction, resulting in small values of *μ*(*s_i_*) and relatively large values of *σ*(*s_i_*) for each node. Moreover, it is not difficult to find that the value of $\bar{U^{c}\left(s_{i}\right)}$ in BASE + DCD_A is only slightly higher than that in BASE, which means that only adjusting the charging direction of the MC without optimizing its staying position cannot significantly improve the charging utility. This is because DCD_A achieves the goal of “shortening the maximum charging duration of nodes within each energy radiation area of an MC” by fine-tuning the charging orientation. However, the degree of improvement in *μ*(*s_i_*) and decrease in *σ*(*s_i_*) of other nodes is limited in this case. In contrast, the average charging utility in BASE + OLSP is relatively high, almost the same as that in FLDC. The reason is that optimizing the location of each staying point effectively shortens the distance between MC and nodes, thereby greatly enhancing the energy receiving efficiency of nodes and shortening the charging duration.

**Figure 10:**
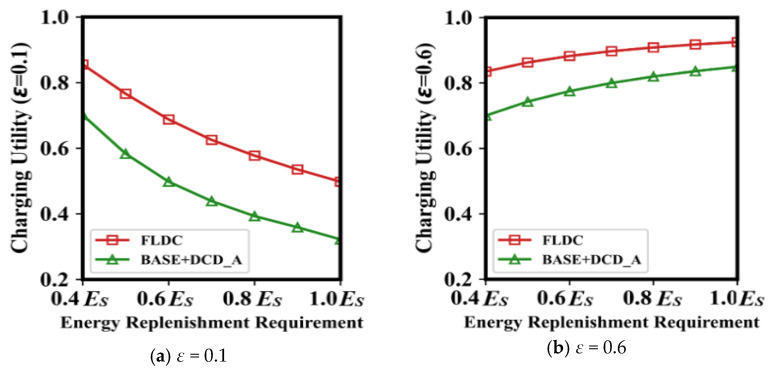
Charging utility with different energy replenishment requirements.

Figure 10 shows the average charging utility with different Energy Replenishment Requirements (ERR). It is known from Figure 9 that regardless of the value of *ε*, the charging utility of BASE is relatively close to that of BASE + DCD_A. Similarly, the difference of $\bar{U^{c}\left(s_{i}\right)}$ between BASE + OLSP and FLDC is also not significant. Therefore, we only show the charging utility of BASE + DCD_A and FLDC in Figure 10 to describe the variation more clearly.

It can be seen from Formula (17) that when *ε* = 0.1, the *U^c^*(*s_i_*) of each node mainly depends on the “duration of a single charge for *s_i_*”, as shown in Figure 10a. In this case, recharging each node to its full battery capacity will undoubtedly result in a low value of $\bar{U^{c}\left(s_{i}\right)}$. As shown in Figure 10a, the average charging utility of nodes in FLDC at this time is only approximately 53.53%, and the value in BASE + DCD_A is even lower at 35.91%. When ERR is reduced, both methods show a significant increase in $\bar{U^{c}\left(s_{i}\right)}$.

In the case that *ε* = 0.6, the average charging utility of a node mainly depends on the “its lifetime that can be extended after being recharged once”, as shown in Figure 10b. When ERR is low, *μ*(*s_i_*) is small, leading to a low value of *U^c^*(*s_i_*). In contrast, a higher ERR results in a larger *μ*(*s_i_*), which enhances the node’s charging utility. In Figure 10b, when the energy replenished to nodes is increased from 40%*E_s_* to *E_s_*, values of $\bar{U^{c}\left(s_{i}\right)}$ in both methods rise by more than 10%.

Furthermore, regardless of the values of *ε* and ERR, the average charging utility in FLDC is always higher than that in BASE + DCD_A (e.g., when *ε* = 0.1 or 0.6, the former is approximately 17% or 9% higher than the latter, respectively), which is due to the OLSP carried out by FLDC.

### 6.2. Impact of θ on Energy Utilization Ratio and Charging Utility

During the process of directional charging, the charging angle of expansion *θ* not only determines the range of energy the MC radiates and the number of nodes it can serve each time, but also affects the energy reception effect of nodes. Therefore, the performance of the system with different values of *θ* is analyzed here. To accurately demonstrate the effect of *θ* on node energy utilization ratio (defined as the proportion of energy received by a node to the energy radiated by the MC for charging it) as well as the average value of node charging utility, the furthest charging distance (*R_c_*) and the energy transmitting power (*p^c^*(*MC*)) of the MC are set to 3.5 m and 10 w, respectively. We mainly show the experimental results in a single NB-VG.

**Figure 11:**
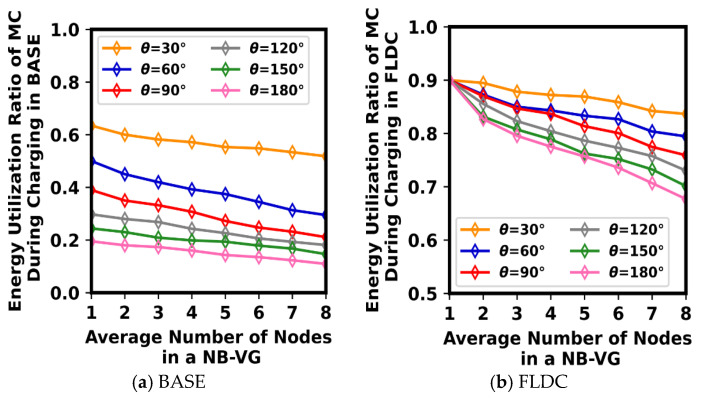
Energy utilization ratio under different values of θ and different numbers of nodes.

As shown in Figure 11a, in the BASE method, regardless of the number of nodes within each NB-VG, a smaller *θ* results in a higher energy utilization ratio for the nodes. This is because when *θ* is small, the deviation between $\vec{CO_{j}}$ and the location of the node being recharged is relatively small. That is to say, *α_ji_* in Formula (1) is small in this case, ensuring that *p^r^*(*s_i_*) can reach a larger value. On the contrary, if *θ* is larger, the energy of the MC is radiated over a wider area, which undoubtedly affects the energy utilization of some nodes located at the edge of this region. As illustrated in Figure 11a, the average energy utilization ratio of the nodes in BASE is only about 20% when *θ* = *π*, which is much lower than that of *θ* = *π*/6 (58–62%).

Figure 11b demonstrates the node energy utilization ratio for different values of *θ* in FLDC. It is evident that when there is only one node in an NB-VG, the value of *θ* does not affect the energy utilization effect of the node, with its average energy utilization ratio reaching up to 90%. According to the algorithm execution process described in Section 5.3, in this case, the MC will adjust its staying position within the NB-VG to the vicinity of the node, almost achieving zero distance-based wireless charging, which results in a higher energy utilization ratio. As the number of nodes in the NB-VG increases, the overall charging utility of all nodes should be taken into account when determining the locations of the MC’s staying points. That is to say, the charging mode will be changed from “zero distance-based one-to-one charging” to “spaced distance-based one-to-many charging”, leading to a decrease in the energy utilization ratio of the nodes. The larger the *θ*, the more pronounced the decrease. However, with the same number of nodes in an NB-VG, the energy utilization ratio of FLDC is higher than that of BASE with each value of *θ.* This indicates the effectiveness of OLSP and DCD_A in our method.

**Figure 12:**
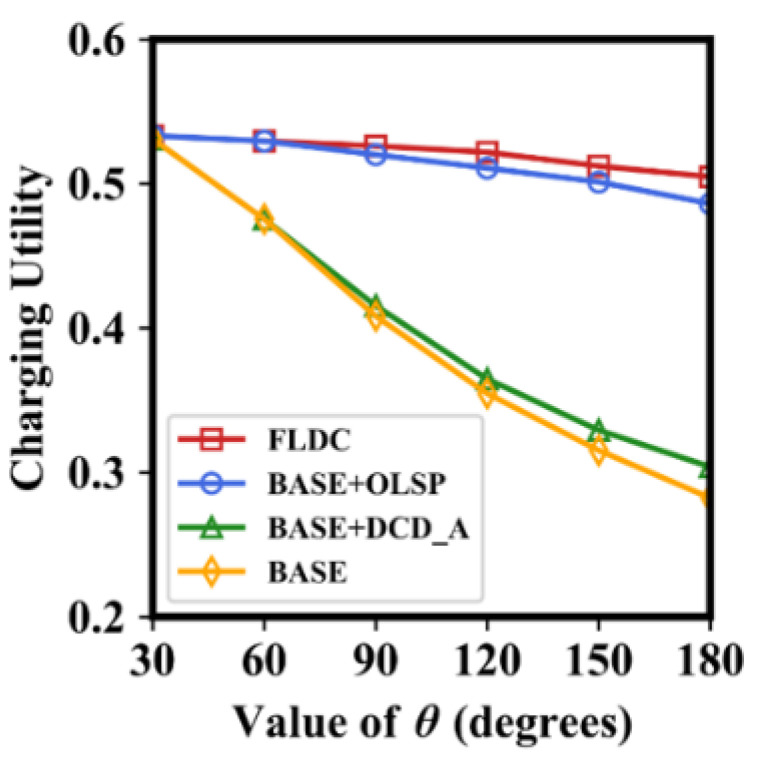
Charging utility with different values of *θ*.

Figure 12 demonstrates the effect of different values of *θ* on the average value of charging utility for the four methods, i.e., BASE, BASE + OLSP, BASE + DCD_A, and FLDC. In order to make the results more distinguishable, we keep the average number of nodes within each NB-VG to two, and the value of *ε* is set to 0.1 (as previously mentioned, the charging utility of nodes is mainly determined by their charging duration). It is easy to see from the figure that when *θ* = *π*/6, the values in all these four methods are high and almost equal to each other. As *θ* increases, the charging utility decreases in each method. It can be seen from Figure 11 that a larger value of *θ* results in a lower average energy utilization ratio of nodes within the MC’s coverage area. To achieve the energy replenishment target, the MC’s charging duration increases in this case, which undoubtedly reduces the charging utility of nodes. However, the charging utility of FLDC and BASE + OLSP decreases relatively slowly, which is also due to the fact that they both optimize the locations of staying points in the gird, thus shortening the node’s charging duration compared to BASE and BASE + DCD_A under the same conditions.

### 6.3. Variation in the Charging Duration of Nodes

**Figure 13:**
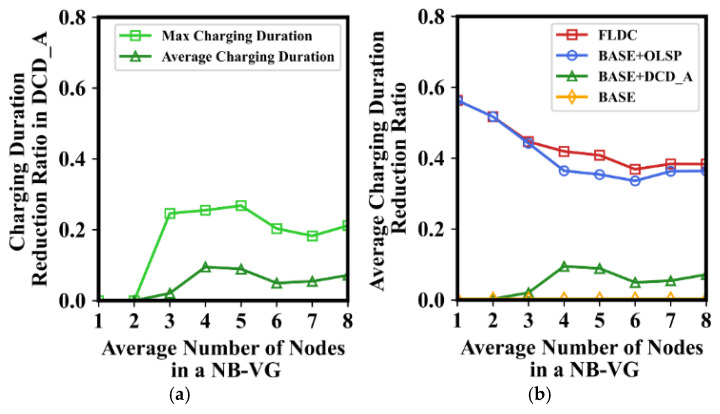
Charging duration of nodes in different methods. (**a**) Charging duration after performing DCD_A; (**b**) charging duration in four methods.

Figure 13a illustrates the charging duration reduction ratio (CDRR for short) for BASE before and after DCD_A have been performed. In order to minimize the impact of nodes with extremely poor positions on simulation results, we randomly initialized the distribution of nodes multiple times and carried out multiple experiments at *θ* = 2π/3 to display the average value of the results. As can be seen from this figure, when the number of nodes in an NB-VG is less than three, the charging duration is not shortened. In this case, the number of nodes that the MC can cover in each energy radiation direction is almost no more than one. Thus, according to the description in Section 5.4, there is no need to perform DCD_A to fine-tune the charging direction of the MC at this time. When the number of nodes ranges from four to eight, the average charging duration is reduced by about 10%. This is due to the fact that the DCD_A realizes the fine-tuning of the charging range by setting the weight coefficients relative to the current charging direction of the MC. This makes its final charging direction slightly biased towards nodes farther away from it, which reduces the difference in the duration that each node is recharged to a certain extent. It can be seen that in the case of five nodes, the value of CDRR has increased by nearly 30%, indicating a significant effect after executing DCD_A.

Figure 13b shows the average charging duration reduction ratio of nodes after performing BASE + OLSP, BASE + DCD_A, and FLDC, compared to the BASE method. The parameter settings are the same as those in Figure 13a, and the results of BASE are also displayed as the benchmark in this figure for ease of comparison. It is not difficult to see that the average value of CDRR after performing BASE + OLSP or FLDC is reduced by about 40–60%, compared to that of BASE. This is because they both optimize the staying position of the MC by performing OLSP, making it closer to nodes. When the number of nodes in an NB-VG ranges from one to three, the effect of this optimization is more significant. As the number of nodes increases, even after optimization, it is unlikely that the staying point is close to each node at the same time. In this case, the value of CDRR in BASE + OLSP and FLDC is reduced (maintained at around 40%). Since BASE + OLSP does not further execute DCD_A, its effect is slightly inferior to that of FLDC.

### 6.4. Performance Comparison between Different Directional Charging Algorithms

**Figure 14:**
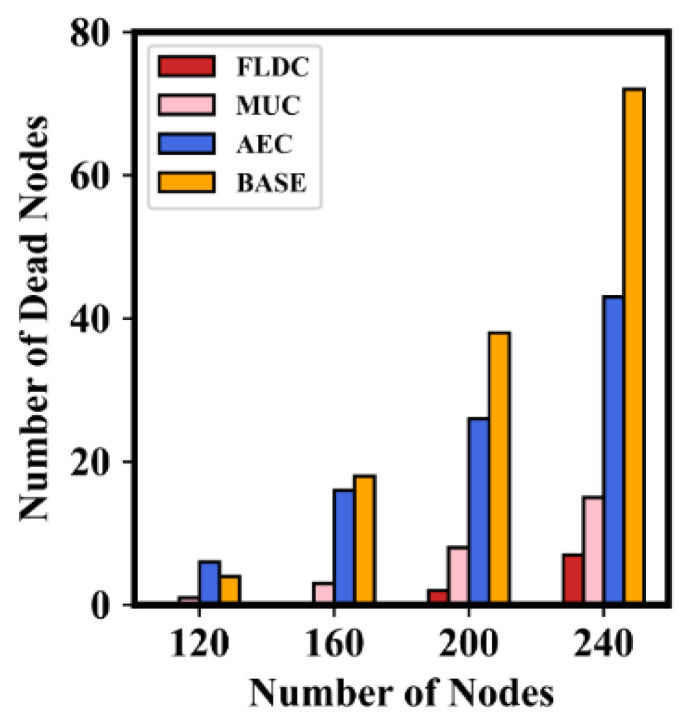
Number of dead nodes in different algorithms.

**Figure 15:**
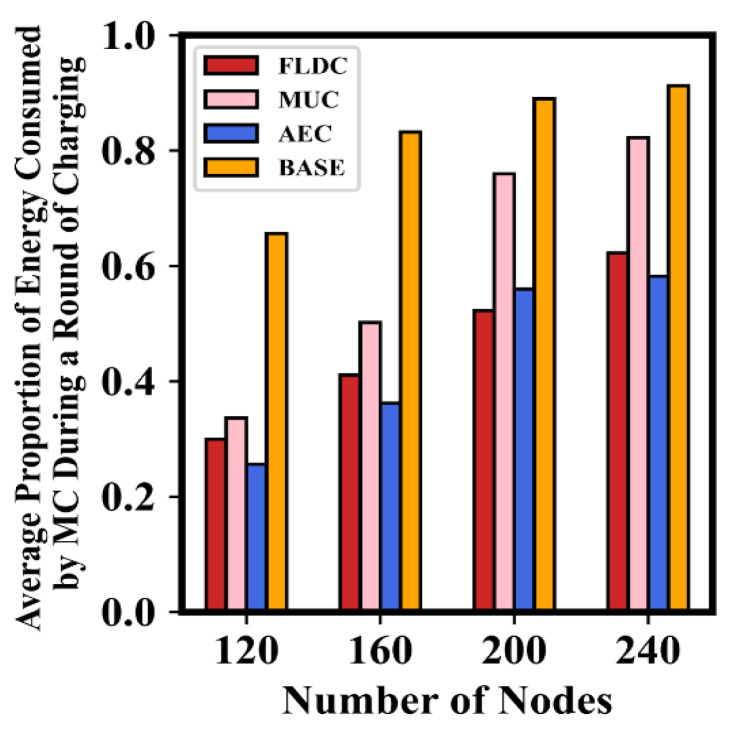
Average proportion of energy consumed by the MC in different algorithms.

**Figure 16:**
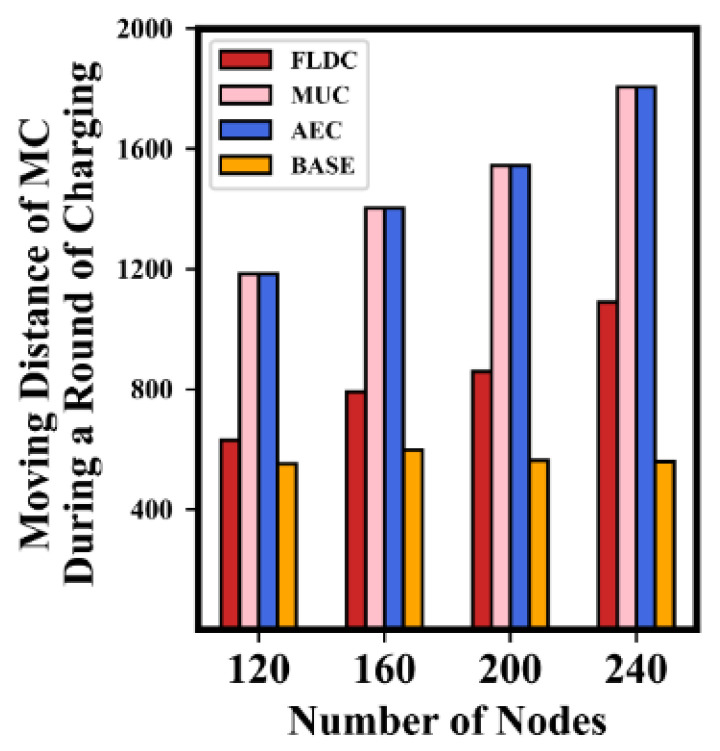
Movement distance of MC during a round of charging.

**Figure 17:**
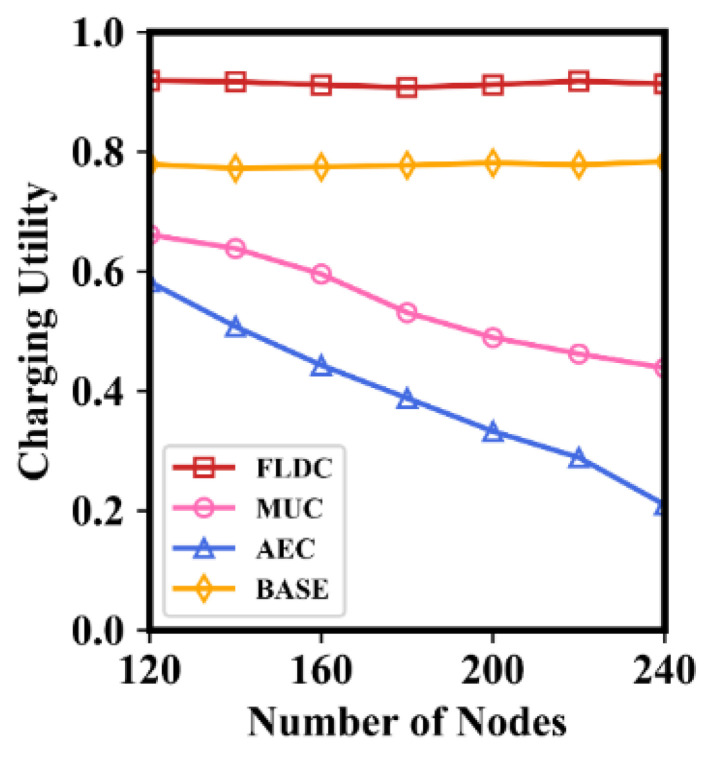
Average charging utility with number of nodes.

In order to further demonstrate the advantages of FLDC, we compare it with AEC, MUC, and BASE in terms of the number of dead nodes, the energy consumption and movement distance of the MC during a round of charging, and the charging utility. Experimental results are shown in Figure 14, Figure 15, Figure 16 and Figure 17.

Figure 14 shows the number of dead nodes in these four methods with different numbers of nodes. Since BASE only sets the traversal order of the MC without further optimizing its staying position and charging direction, there is a high number of dead nodes in this method. On the contrary, FLDC further performs OLSP and DCD_A on the basis of BASE, ensuring a high survival rate of nodes (almost no nodes die when the number of them is 120 or 160). In addition, both AEC and MUC take the node to be replenished as the staying point, and they construct the “set of nodes to be recharged” successively with the purpose of “maximizing the overall charging utility”. Therefore, compared with BASE, the staying points in these two methods are closer to nodes to be recharged, which makes the number of dead nodes lower than that of BASE. However, unlike our method that uses a two-layer fuzzy logic-based approach to set the grid priority and the traversal order of the MC, in AEC and MUC, the MC always traverses the staying points along the shortest path and the charging duration of each round is the same. This may result in some nodes with low residual energies dying since they cannot be recharged in time. For this reason, the number of dead nodes in AEC and MUC is higher than that in FLDC, and the difference is more pronounced when the number of nodes is higher. It should be pointed out that for ease of execution, the staying duration of the MC in each “set of nodes to be recharged” is set to the same value in AEC, which is obviously unable to satisfy the nodes’ different energy replenishment needs. On the other hand, the MUC method reasonably allocates the staying duration of the MC in set, so its number of dead nodes is lower than that of AEC.

Figure 15 verifies the energy consumption of the MC after completing a round of service using each algorithm. For the sake of comparison, normalization is performed here to only show the proportion of the average energy consumed by the MC in a single round to its full energy capacity *E_MC_* (hereafter referred to as the “energy consumption proportion”). It is not difficult to see that in BASE, the staying point of the MC in each NB-VG is always its geometric center. In this case, it often requires more energy to supply nodes far away from it and most of the energy dissipates in free space. Thus, the energy consumption proportion of the MC is highest in BASE. By contrast, this value in FLDC is significantly lower than that of BASE. Even in the case of a large number of nodes in the network (e.g., 240), the proportion is only 62.3%, which ensures that the MC can serve more nodes during a round of charging and also indicates that FLDC can better adapt to the expansion of network scale.

Moreover, it is easy to see that the energy consumption proportion of the MC in AEC is also low. It is even lower than that of FLDC when the number of nodes is 120, 160 or 240. As mentioned earlier, the staying duration of the MC in each “set of nodes to be recharged” is the same in the AEC method, regardless of the energy replenishment requirement of nodes. Therefore, with a fixed duration on recharging for a single round of traversal, the proportion of energy consumption of the MC in AEC is not very high. However, this can easily cause some nodes to become exhausted in the near future due to insufficient energy supply. MUC, in contrast, adjusts the duration of the MC’s stay at each point to meet the different charging demand of nodes as much as possible. Thus, the energy consumption proportion of the MC in this method is higher than that of AEC. As the number of nodes increases, the MC in MCU will be more inclined to meet the demand of nodes with more energy requirement. In this case, its energy consumption proportion is higher (e.g., in a network of 240 nodes, the value reaches up to 82.2%, which is almost close to the result of BASE (91.3%)).

The average movement distance of the MC to complete a round of charging tasks is shown in Figure 16. It can be seen that the value of this term in AEC and MUC is always the same and much higher than that in BASE and FLDC. This is because AEC and MUC consider all nodes as the “objects waiting to be recharged”, that is, the “set of nodes to be recharged” they construct includes all nodes in the network. Moreover, the MC always tries to traverse each set along a fixed path, so the length of the path in these two methods is equal to each other. According to Section 5.2, it can be seen that the MC in BASE and FLDC only needs to serve those nodes whose residual lifetime are less than 2*T_max_*. This means it only needs to traverse a portion of grids, resulting in a shorter traveling distance.

It is worth noting that when the total number of nodes is 120, the simulation results of BASE and FLDC are relatively close to each other. However, as the number of nodes increases, the number of grids that need to be traversed by the MC also rises, so the movement distance of the MC in FLDC becomes longer. As for BASE, the MC is likely to have no extra energy left to serve more nodes at this point (e.g., as can be seen in Figure 15, the proportion of energy consumed by the MC in BASE was close to 90% when the number of nodes reaches 200). As a result, the gap between the simulation results of BASE and FLDC increases when the number of nodes is high.

Figure 17 demonstrates the variation of charging utility with the number of nodes when *ε =* 0.6. To show the fairness of this comparison, the charging utility after carrying out AEC and MUC is also calculated using Formula (17). Simulation results show that the charging utility in FLDC or BASE is significantly higher than the other two methods and is not affected by the number of nodes. This is because, after obtaining the grid priority with the help of the two-layer fuzzy logic system, the MC in both FLDC and BASE can supply more energy to nodes, which results in a larger value of *μ*(*s_i_*), thus ensuring a higher charging utility. After executing OLSP and DCD_A, the average charging duration of FLDC is less than that of BASE, so the charging utility of the former is slightly higher (by about 13%) than the latter.

Due to the fact that AEC and MUC are not like FLDC and BASE in which the MC only charges nodes with shorter residual lifetime, their charging utility is relatively low. Furthermore, the average charging utility of AEC is lower than that of MUC. This is because the stay duration of the MC is always fixed in AEC, which is unable to adapt to different charging demands of nodes as in MUC. In addition, there are more and more “sets of nodes to be recharged” in AEC and MUC as the number of nodes increases. Nevertheless, the charging duration is always fixed in these two methods, which may lower the value of *μ*(*s_i_*). Thus, the average charging utility of AEC and MUC decreases as the number of nodes increases.

## 7. Conclusions

In this paper, we propose a fuzzy logic-based directional charging scheduling scheme to improve the charging efficiency in WRSNs. Firstly, the charging areas for the MC at each staying point are determined by dividing the network into hexagons. Next, the priority of nodes as well as hexagons is dynamically calculated every time to enable the MC to select the next staying point in order. To minimize the sum of the charging distance, a method for optimization of the location of staying points is proposed. Finally, the charging directions of the MC at each staying point are fine-tuned to balance the charging duration of nodes in the same coverage set. Simulation results show that the proposed method can effectively improve the charging utility as well as the survival rate of nodes compared with other methods of the same type.

Considering the fact that the attributes of sensing nodes are not necessarily fixed in practical situations, in our future work, we will consider dynamic changes of sensing nodes, such as the movement of node position, the change of node energy consumption rate with sensing events, etc., so that our algorithms can be more closely matched with practical applications. We will also consider adding new nodes to the charging queue to improve the energy utilization if the MC capacity is sufficient during a round of charging.

## Figures and Tables

**Table 1:** Comparison of the related works.

	Periodic Charging	On-Demand Charging	Omnidirectional Charging	Directional Charging
[18]	Yes	No	-	-
PSBLA [19]	Yes	No	-	-
GACS [20]	Yes	No	-	-
PDESM [21]	Yes	No	-	-
DFWA [22]	Yes	No	Yes	No
Q-charging [23]	No	Yes	Yes	No
JOLOT [24]	No	Yes	-	-
TSCA [25]	No	Yes	-	-
DWDP [26]	No	Yes	-	-
FLCSD [27]	No	Yes	Yes	No
MTCS [28]	No	Yes	Yes	No
[29]	No	Yes	Yes	No
APPRO [30]	No	Yes	Yes	No
AD-WPT [31]	-	-	No	Yes
EEADC [32]	No	Yes	No	Yes
MRSDs [33]	-	-	No	Yes
MTCD [34]	-	-	Yes	Yes
AEC, MUC [35]	Yes	No	No	Yes

**Table 2:** Parameters of the network.

Symbol	Definition	Unit
*N*	number of nodes	-
*s_i_*	rechargeable sensor node	-
*L*	network length	m
*W*	network width	m
*E_s_*	full battery capacity of node	mAH
*N_MC_*	number of MCs	*-*
*E_MC_*	full battery capacity of MC	mAH
*R_c_*	the farthest reachable charging distance	m
*θ*	charging angle of expansion	*-*
*p^r^*(*s_i_*)	energy receiving power of *s_i_*	w
*d*(*MC_j_*, *s_i_*)	distance between *MC_j_* and *s_i_*	m
*η*	energy receiving power coefficient of node in “zero distance-based wireless recharging” mode	-

**Table 3:** Parameters mentioned in Section 4.

Symbol	Definition	Unit
*E^c^*(*NB-VG_k_*)	energy consumed by the MC for charging all nodes in NB-VG_k_	mAH
*NoN*(*NB-VG_k_*)	number of nodes in NB-VG_k_	*-*
*p^c^*(*MC*)	energy transmission power (charging power) of the MC	w
*E^c^_max_*(*NB-VG*)	the maximum energy consumed by the MC to charge all NB-nodes during a round of service time	mAH
*Num*(*NB-VG*)	number of NB-VGs which have nodes in need of recharging	-
*E^c^*(*B-VG_k_*)	energy consumed by the MC to charge all nodes in B-VG_k_	mAH
*NoN*(*B-VG_k_*)	number of nodes in B-VG_k_	*-*
*E^c^_max_*(*B-VG*)	the maximum energy consumed by the MC to charge all B-nodes during a round of service time	mAH
*Num*(*B-VG*)	number of B-VGs which have nodes in need of recharging	*-*
*E^m^*(*NB-VG*)	energy consumed by the MC on moving during a round of charging for nodes in all the NB-VGs	mAH
$\bar{d_{VG}}$	average distance that the MC moves between two adjacent grids	m
*e_m_*	energy consumption of the MC on moving per unit of distance	w
*E^m^*(*B-VG*)	energy consumed by the MC on moving during a round of charging for nodes in all the B-VGs	mAH
*d_B-VG_*(*i*, *i *+ 1)	distance between two adjacent B-nodes (i.e., *s_i_* and *s_i+_*_1_) that are sequentially traversed by the MC	m
*T_max_*	maximum duration for a single MC to finish a round of service	s
*T^c^_max_*(*NB-VG*)	the upper limit values of the duration for an MC to charge all nodes in an NB-VG	s
*T^c^_max_*(*B-VG*)	the upper limit values of the duration for an MC to charge all nodes in a B-VG	s
*T^m^*	the time an MC spends on moving during a round of service	s
$\bar{d\left(MC_{j},s_{i}\right)}$	the mean distance between all NB-nodes and the center of the NB-VG they are located in	m
$\bar{n_{N\text{B-VG}}}$	average number of nodes in each NB-VG	-
*v*	movement speed of the MC	m/s
*p*(*s_i_*)	energy consumption rate of *s_i_*	w
*t^r^*(*s_i_*)	residual lifetime of *s_i_*	s
*t^w^*(*s_i_*)	the active duration of *s_i_* from the last time it has been recharged to the current moment	s

**Table 4:** Linguistic value and ranges of membership functions of the first layer.

Input	Linguistic Value	Range of Membership Function
*t^r^*(*s_i_*)	Low	[0, 0, 0.15 × 2*T*, 0.35 × 2*T*]
	Medium	[0.15 × 2*T*, 0.35 × 2*T*, 0.65 × 2*T*, 0.75 × 2*T*]
	High	[0.65 × 2*T*, 0.85 × 2*T*, 2*T*, 2*T*]
*p*(*s_i_*)	Low	[0, 0, 0.15 × max(*p*(*s_i_*)), 0.35 × max(*p*(*s_i_*))]
	Medium	[0.15 × max(*p*(*s_i_*)), 0.35 × max(*p*(*s_i_*)), 0.65 × max(*p*(*s_i_*)), 0.85 × max(*p*(*s_i_*))]
	High	[0.65 × max(*p*(*s_i_*)), 0.85 × max(*p*(*s_i_*)), max(*p*(*s_i_*)), max(*p*(*s_i_*))]
*d*(*s_i_*, *center_k_*)	Near	[0, 0, 0.15 × *R_c_*, 0.375 × *R_c_*]
	Medium	[0.15 × *R_c_*, 0.375 × *R_c_*, 0.6 × *R_c_*]
	Far	[0.375 × *R_c_*, 0.6 × *R_c_*, *R_c_*, *R_c_*]

**Table 5:** Output value of the first layer.

Output	Linguistic Value	Range of Membership Function
Node Priority (NP)	Very Low	[0, 0, 0.15, 0.2]
	Low	[0.15, 0.2, 0.35, 0.4]
	Medium	[0.35, 0.4, 0.6, 0.65]
	High	[0.6, 0.65, 0.8, 0.85]
	Very High	[0.8, 0.85, 1, 1]

**Table 6:** Fuzzy inference rules for the first layer.

Rule	Input			Output	Rule	Input			Output	Rule	Input			Output
	*t^r^*(●)	*p*(●)	*d*(●)	NP		*t^r^*(●)	*p*(●)	*d*(●)	NP		*t^r^*(●)	*p*(●)	*d*(●)	NP
R1	L	L	N	M	R10	M	L	N	L	R19	H	L	N	VL
R2	L	L	M	M	R11	M	L	M	L	R20	H	L	M	VL
R3	L	L	F	M	R12	M	L	F	L	R21	H	L	F	VL
R4	L	M	N	H	R13	M	M	N	M	R22	H	M	N	L
R5	L	M	M	H	R14	M	M	M	M	R23	H	M	M	L
R6	L	M	F	H	R15	M	M	F	M	R24	H	M	F	L
R7	L	H	N	VH	R16	M	H	N	H	R25	H	H	N	M
R8	L	H	M	VH	R17	M	H	M	H	R26	H	H	M	M
R9	L	H	F	VH	R18	M	H	F	H	R27	H	H	F	M

**Table 7:** Linguistic values and ranges of membership functions of the second layer.

Input	Linguistic Value	Range of Membership Function
*NP*(*s_i_*)	Very Low	[0, 0, 0.15 × max(*NP*(*s_i_*)), 0.2 × max(*NP*(*s_i_*))]
	Low	[0.15 × max(*NP*(*s_i_*)), 0.2 × max(*NP*(*s_i_*)), 0.35 × max(*NP*(*s_i_*)), 0.4max(*NP*(*s_i_*))]
	Medium	[0.35 × max(*NP*(*s_i_*)), 0.4 × max(*NP*(*s_i_*)), 0.6 × max(*NP*(*s_i_*)), 0.65 × max(*NP*(*s_i_*))]
	High	[0.6 × max(*NP*(*s_i_*)), 0.65 × max(*NP*(*s_i_*)), 0.8 × max(*NP*(*s_i_*)), 0.85 × max(*NP*(*s_i_*)),]
	Very High	[0.8 × max(*NP*(*s_i_*)), 0.85 × max(*NP*(*s_i_*)), max(*NP*(*s_i_*)), max(*NP*(*s_i_*))]
*d*(*center_k_*, *MC_j_*)	Near	[0, 0, 0.3 × max(*d*(*center_k_*, *MC_j_*)), 0.6 × max(*d*(*center_k_*, *MC_j_*))]
	Medium	[0.3 × max(*d*(*center_k_*, *MC_j_*)), 0.6 × max(*d*(*center_k_*, *MC_j_*)), 0.9 × max(*d*(*center_k_*, *MC_j_*))]
	Far	[0.6 × max(*d*(*center_k_*, *MC_j_*)), 0.9 × max(*d*(*center_k_*, *MC_j_*)), max(*d*(*center_k_*, *MC_j_*)), max(*d*(*center_k_*, *MC_j_*))]

**Table 8:** Output value of the second layer.

Output	Linguistic Value	Range of Membership Function
Grid Priority (GP)	Very Low	[0, 0, 0.15, 0.2]
	Low	[0.15, 0.2, 0.35, 0.4]
	Medium	[0.35, 0.4, 0.6, 0.65]
	High	[0.6, 0.65, 0.8, 0.85]
	Very High	[0.8, 0.85, 1, 1]

**Table 9:** Fuzzy inference rules for the second layer.

Rule	Input		Output	Rule	Input		Output	Rule	Input		Output
	*d*(●)	*NP*(●)	GP		*d*(●)	*NP*(●)	GP		*d*(●)	*NP*(●)	GP
R1	N	VL	L	R6	M	VL	VL	R11	F	VL	VL
R2	N	L	M	R7	M	L	M	R12	F	L	L
R3	N	M	H	R8	M	M	M	R13	F	M	L
R4	N	H	H	R9	M	H	M	R14	F	H	M
R5	N	VH	VH	R10	M	VH	VH	R15	F	VH	H

**Table 10:** Simulation parameters.

Symbol	Definition	Value
*L*	Network length	120 m
*M*	Network width	100 m
*R_c_*	The maximum charging distance of the MC	2.7 m
*E_s_*	Full energy capacity of node	500 mAH
*E_MC_*	Full energy capacity of the MC	3500 mAH
*p^c^*(*MC*)	Energy transmission power of the MC (Charging power)	5 w
*e_m_*	Energy consumption of the MC on moving per unit distance	8 w
*v*	Movement speed of the MC	2 m/s
*p*(*s_i_*)	Energy consumption rate of *s_i_*	0.1 w–0.9 w
*c*	Parameters for calculating the energy receiving power of node	0.1161
*μ*	Parameters for calculating the energy receiving power of node	3.893
*Β*	Parameters for calculating the energy receiving power of node	0.1
*λ*	Coefficient of energy receiving power of the node for zero distance-based charging	0.9

## Data Availability

Data is contained within the article.
